# Derivatives of Tenuazonic Acid as Potential New Multi-Target Anti-Alzheimer’s Disease Agents

**DOI:** 10.3390/biom11010111

**Published:** 2021-01-15

**Authors:** Viviana Poliseno, Sílvia Chaves, Leonardo Brunetti, Fulvio Loiodice, Antonio Carrieri, Antonio Laghezza, Paolo Tortorella, João D. Magalhães, Sandra M. Cardoso, M. Amélia Santos, Luca Piemontese

**Affiliations:** 1 Department of Pharmacy and Pharmaceutical Sciences, University of Bari “A. Moro”, via E. Orabona 4, 70125 Bari, Italy; v.poliseno1@studenti.uniba.it (V.P.); leonardo.brunetti@uniba.it (L.B.); fulvio.loiodice@uniba.it (F.L.); antonio.carrieri@uniba.it (A.C.); antonio.laghezza@uniba.it (A.L.); paolo.tortorella@uniba.it (P.T.); 2 Centro de Química Estrutural and Departamento de Engenharia Química, Instituto Superior Técnico, Universidade de Lisboa, Av. Rovisco Pais, 1049-001 Lisboa, Portugal; silvia.chaves@tecnico.ulisboa.pt; 3 CNC—Center for Neuroscience and Cell Biology, University of Coimbra, 3004-504 Coimbra, Portugal; joaoduartemagalhaes7@gmail.com (J.D.M.); cardoso.sandra.m@gmail.com (S.M.C.); 4 Institute of Molecular and Cell Biology, Faculty of Medicine, University of Coimbra, 3000-548 Coimbra, Portugal

**Keywords:** Alzheimer’s disease, tenuazonic acid, donepezil, neurodegenerative, metal chelation, multifunctional drugs, acetylcholinesterase, antioxidant, amyloid

## Abstract

Alzheimer’s disease (AD) is generally recognized as a multifactorial neurodegenerative pathology with an increasing impact on society. Tenuazonic acid (TA) is a natural compound that was recently identified as a potential multitarget ligand with anti-cholinesterase, anti-amyloidogenic and antioxidant activities. Using its structure as a chemical scaffold, we synthesized and evaluated new derivatives (1–5), including tenuazonic-donepezil (TA-DNP) hybrids (4 and 5) due to the clinical importance of the anti-AD drug donepezil. These novel compounds all achieved activity in the micromolar range towards all selected targets and demonstrated to be potentially orally absorbed. Moreover, a selected compound (1) was further investigated as a chelating agent towards copper (II), zinc (II) and iron (III) and showed good chelating ability (pFe = 16.6, pCu = 11.6, pZn = 6.0 at pH 7.4). Therefore, the TA motif can be considered an interesting building block in the search for innovative multi-functional anti-neurodegenerative drugs, as exemplified by hybrid 5, a promising non-cytotoxic lead compound adequate for the early stages of AD, and capable of ameliorating the oxidative status of SH-SY5Y human neuroblastoma cells.

## 1. Introduction

**Figure 1:**
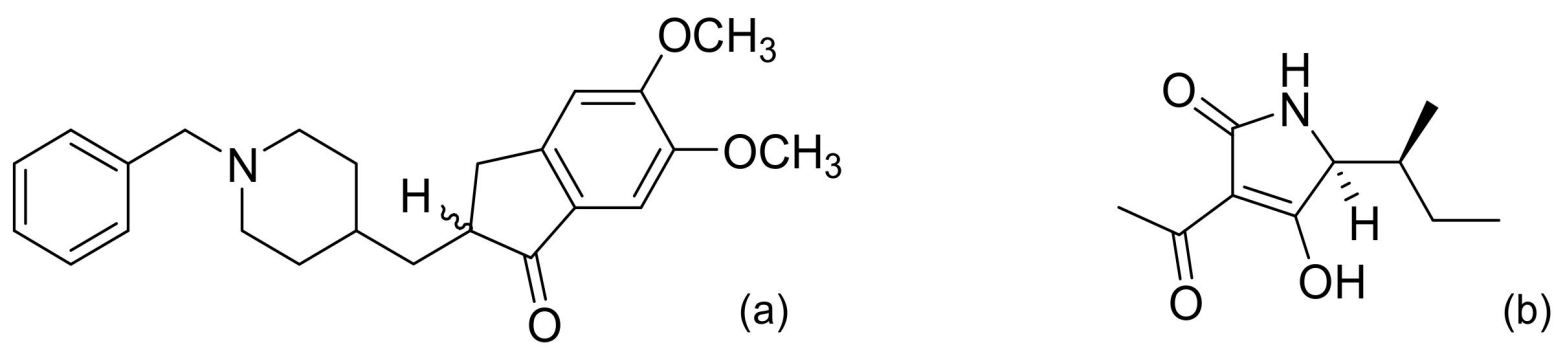
(**a**) Donepezil (DNP); (**b**) Tenuazonic acid (TA).

Alzheimer’s disease (AD) is one of the most common neurodegenerative diseases, characterized by a drastic and progressive decline in memory and cognitive abilities [1]. It represents the main cause of dementia worldwide, and it is also recognized as a socio-economic problem especially due to the constant care needed by patients and their families [2]. The number of people affected by AD is substantial, reaching 46 million globally, and is poised to rise sharply in the coming decades, as a result of increasing life expectancies worldwide [3]. No effective treatments are currently available for this form of dementia, despite billions of dollars having been invested towards this goal, the United States being the single world region responsible for most clinical trials [4]. Only five symptomatic drugs were approved in the EU and US, four acetylcholine esterase (AChE) inhibitors (tacrine, donepezil, Figure 1a, rivastigmine and galantamine), and memantine, an antagonist of the NMDA receptor [2].

The etiology of neurodegeneration in AD is complex, and it is now clear that it involves a plethora of factors which must be tackled at the same time in order to achieve a valid therapeutic effect. These factors include a reduced level of acetylcholine (ACh), the formation of extracellular aggregates of amyloid β (Aβ) and an overproduction of free radicals and reactive oxygen species (ROS) resulting in oxidative stress. Dyshomeostasis of metal ions, particularly Cu^2+^, Zn^2+^ and Fe^3+^, is thought to be responsible for the precipitation of Aβ and for the onset of neuroinflammatory processes which contribute to oxidative stress [5,6,7]. In accordance with this multifaceted nature, numerous researchers have focused on the development of drugs aiming at potential one-molecules-several-target therapies, including AChE inhibition, metal chelation and antioxidant effects [2,8,9,10,11]. In the last few years, our research group has made several discoveries regarding potential scaffolds for the development of multi-target agents, resulting in compounds with interesting activity profiles, especially in regards to AChE inhibition and metal chelation [12,13,14]. Following the multi-target approach, one innovative hypothesis that could lead to a future successful therapy entails the correlation between metabolic function (or rather dysfunction) of neurons and glial cells and neurodegeneration during the progression of AD [15,16,17,18,19,20]. 

In this context, natural compounds play an important role in the development of novel multitarget agents for the treatment of many pathologies, including AD, and have been garnering increasing attention in the last few years [17,18]. Furthermore, plant-derived polyphenols and other natural molecules such as fungal metabolites [19] may also prevent neuronal damage due to their metal chelating action, reducing the aggregation of Aβ into amyloid plaques and also the formation of ROS [20]. Semisynthetic and fully synthetic derivatives of such natural compounds have been taken into consideration as potential drug candidates [21,22].

In a recent study, we selected six natural compounds on the basis of their chemical structures (tenuazonic acid, epi-radicinol, mycophenolic acid, 6-methoxymellein, radicinin, visoltricin/fungerin). These compounds were tested against a series of targets involved in AD onset. Thus, some of them showed a multi-target activity profile, which allowed us to consider them as starting hit compounds for the development of future potential anti AD-drugs [19].

Tenuazonic acid (TA, Figure 1b) was the most interesting scaffold for the development of new potential drugs for the treatment of AD due to its properties as inhibitor of AChE and as an antioxidant, as well as for its metal chelation capacity [19].

**Figure 2:**
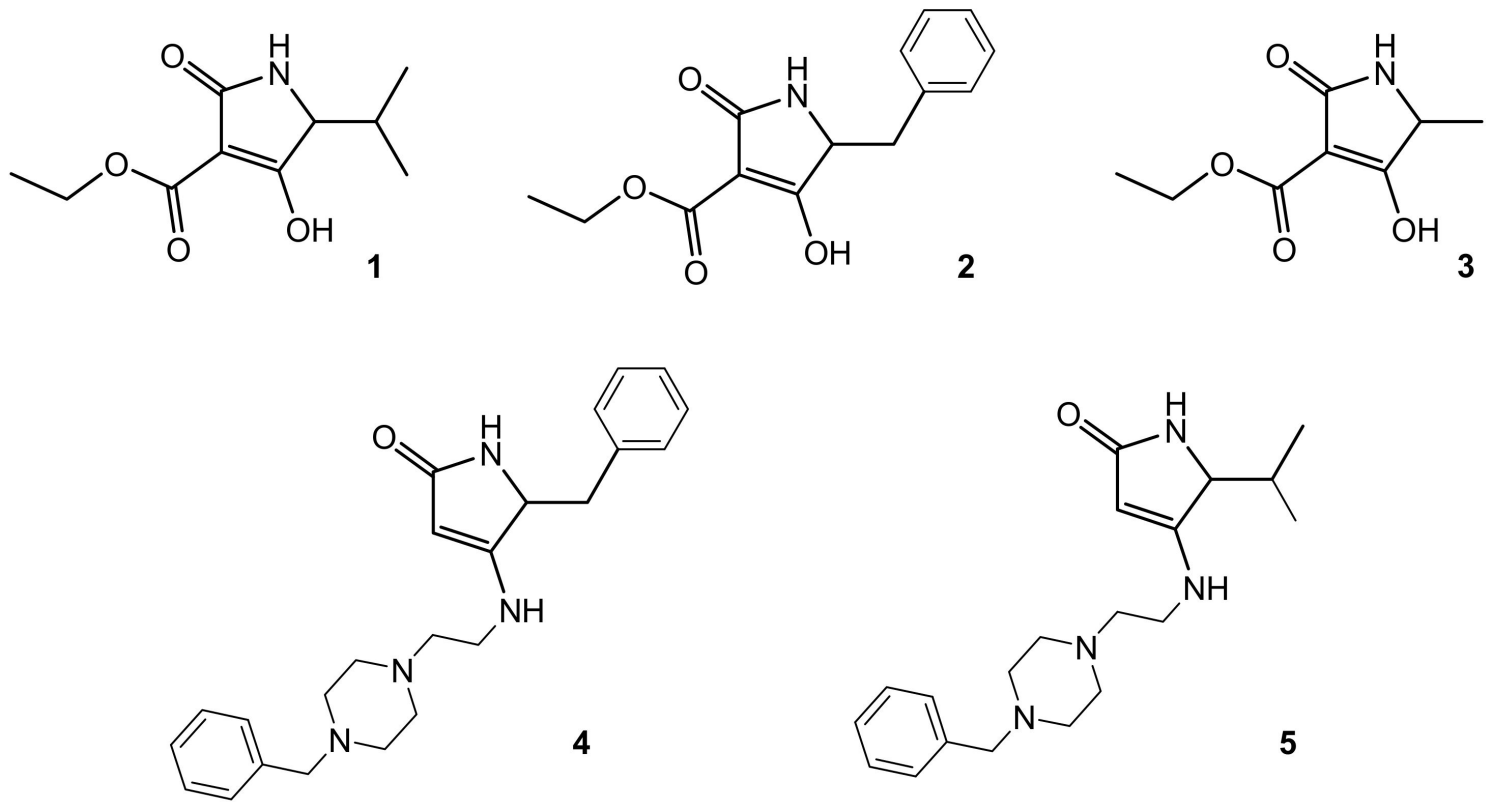
Structural formula of the developed compounds.

The herein developed compounds (Figure 2) were designed by structural modification of the TA scaffold (compounds **1**–**3**) or by hybridization with a donepezil-mimetic-moiety (compounds **4** and **5**, as tenuazonic-donepezil (TA-DNP) hybrids) trying, in the latter case, to improve inhibitory activity towards AChE as well: the inclusion of the DNP-like benzylpiperazine moiety should optimize the interactions formed by the inhibitors with the enzyme pocket, allowing the occupation of the catalytic active site (CAS). Moreover, in order to characterize their biological activity, our novel compounds were assayed for acetylcholine esterase inhibition, antioxidant activity, inhibition of Aβ self-aggregation, cell viability and neuroprotection. It was also interesting to investigate the chelating capacity of these compounds towards Fe(III), Cu(II), Zn(II): thus, compound **1** was chosen as a model to determine this potential activity.

## 2. Results and Discussion

### 2.1. Chemistry

**Scheme 1:**
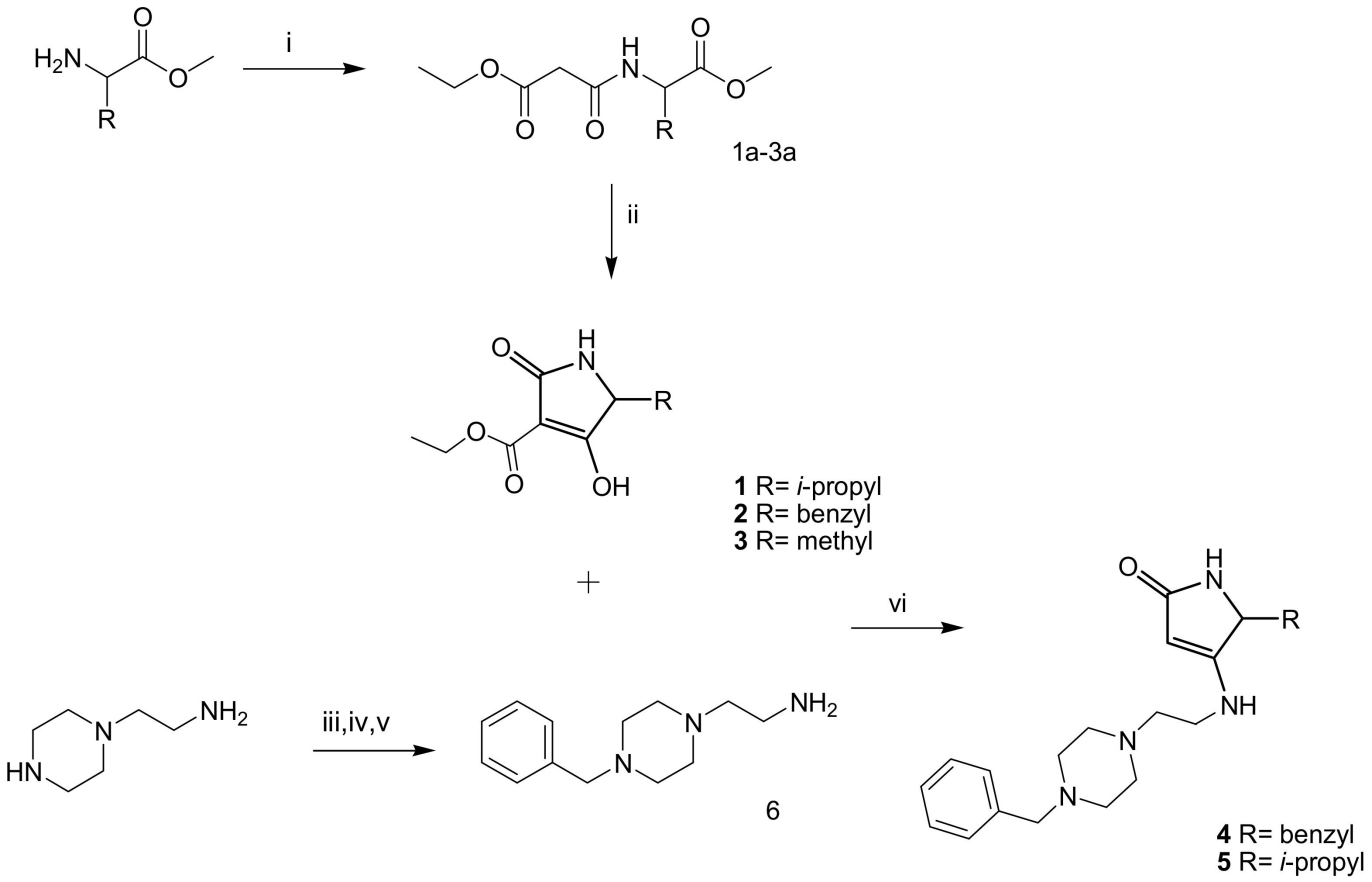
Reagents and conditions: (i) ethyl malonyl chloride, triethylamine, anhydrous CH_2_Cl_2_, N_2_, 0 °C, 24 h; (ii) Na, abs. ethanol, reflux 4 h; (iii) phthalic anhydride, 160 °C, 4 h; (iv) benzyl bromide, KOH, 96° ethanol, 24 h RT; (v) aq solution of MeNH_2_ (40% *w*/*w*), 60 h, RT; (vi) **1** or **2**, THF, 5 h, reflux.

Tenuazonic (TA) congeners and Tenuazonic-Donepezil (TA-DNP) hybrids were synthesized as racemic mixtures according to Scheme 1. In particular, starting from commercial aminoacids and ethyl malonyl chloride, we have synthesized the linear intermediates **1a**–**3a**, which were subsequently cyclized in presence of sodium ethoxide under reflux to afford **1**–**3** [23,24,25,26,27,28]. Compounds **4** and **5** were obtained through reaction of **1** and **2**, respectively, with the crucial intermediate 6 [13,23,29]. The intermediate 6 was synthesized starting from the commercial *N*-aminoethylpiperazine as previously reported [12,29]. 

### 2.2. AChE Inhibition, Radical Scavenging Activity and Inhibition of Aβ_1-42_ Aggregation

Important biological assays, as well as measurements of radical scavenging activity, were performed on the newly synthesized compounds, whose results are depicted in Table 1. In particular, the classical panel of tests (AChE inhibition, radical scavenging activity and inhibition of Aβ1-42 aggregation, with or without bivalent copper) was useful to explore the possible structure-activity relationship of tenuazonic acid (TA) derivatives and of tenuazonic acid—donepezil(TA-DNP) hybrids. 

All new racemic compounds were tested as acetylcholinesterase inhibitors via an in vitro assay, following a modification of Ellman’s method [30] in three independent experiments, using tacrine as a reference compound. These biological activities were also referred to tenuazonic acid and donepezil, included in the results table (Table 1) for comparison purposes. TA was previously assayed for the inhibition of electric eel AChE, but in slightly different conditions, obtaining an IC_50_ of 8.13 ± 0.08 μM [19]. The IC_50_ values of AChE inhibitory activity obtained for the TA analogues (**1**–**3**) are slightly better, but comparable with those obtained for the parent compound (TA) in the same experimental conditions (65 ± 4 μM).

Interestingly, but not surprisingly, the TA-DNP hybrids (**4** and **5**) showed enhanced AChE inhibitory profiles. This can be attributed with certainty to the benzylpiperazine moiety (DNP pharmacophore derivative), due to the important and well-known binding interaction within the active site (cationic active site, CAS) of the enzyme [12,13,14]. As expected, compounds **4** and **5** are less effective than donepezil, probably due to the absence of the fused dimethoxybenzene ring of the DNP indanone moiety, and concomitantly lower interactions with the peripheral anionic site (PAS) of the enzyme. Furthermore, multi-target ligands are very often less active than compounds with single activities, since the presence of multiple pharmacophores makes ligand optimization much more difficult.

Since some of the compounds (**1–3**) bear an enolic hydroxyl group, their antioxidant activity was anticipated, namely because the weak OH bond can favor free radical inactivation. Therefore, they were assayed for radical scavenging activity using the DPPH method. The results are consistent with this assumption, considering the moderate antioxidant activity showed in the tested conditions, at least for compounds **1** and **2** (Table 1).

The anti-amyloidogenic activity of all the compounds was also evaluated in vitro, in the absence and in presence of Cu(II), by measuring the fluorescence emission of Thioflavin T (ThT) [31]. Inhibition of Cu^2+^-induced Aβ aggregates by **4** and **5** was not performed since these compounds do not contain a metal chelating moiety (see below). The Aβ self-aggregation inhibitory activity shown by all compounds is higher than that of parent compound TA, with the exception of compound **2** (probably due to solubility issues at the experimental conditions of the assays). The fact that the reference value previously reported by us for TA (50 ± 8%) [19] was obtained for quite different conditions, including a much higher inhibitor concentration (100 μM instead of 40 μM) is noteworthy. TA was also preliminarily tested for inhibition of Cu^2+^-induced Aβ aggregates but did not demonstrate a significant difference of effect (data not shown). This may be due to different electronic contributions between the ketone and the ester functions. Therefore, the ester derivatives seem to be better chelators compared to TA. 

It is important to note that the increase in inhibitory potential in the case of the TA-DNP hybrids, apparently confirms that the extra benzylpiperazine moieties of compounds **4** and **5**, can interfere with the formation of Aβ sheets by enabling further interactions with the peptide. Moreover, the chelating compounds **1**–**3** also present a relatively higher inhibition of Aβ aggregation in the presence of copper, in accordance with the fact that they are copper chelators with only average activity (see Section 2.3). 

The results contained in Table 1 and those in Section 2.3. suggest that the inhibition of Aβ aggregation must be mainly explained by ligand intercalation between the β-sheets of Aβ fibrils, as previously found for other compounds [12,13,14], and not by competition for copper between the compounds and Aβ peptide. In fact, the conditional dissociation constant (*K*’_d_) for the copper complex of **1** calculated at pH 7.4 is 83 μM, much higher than the proposed range (*K*’_d_ = 1–10 picomolar) corresponding to chelators able to retrieve copper from the Aβ peptide (*K*’_d_ = 10 picomolar–100 nanomolar for Cu(Aβ) complexes) [32].

Aiming to gain proper indications on how the TA derivatives’ absolute configuration could affect their binding properties, and to challenge at the same time any molecular similarity with known anti-Alzheimer agents, dockings of the most interesting derivative were enrolled for both the (*R*) and (*S*) configuration of compound **5** using the eeAChE complex with the (*R*) enantiomer of donepezil (PDB entry 1EVE) [33]. As a first but crucial step, a blind docking carried out on the whole receptor surface was performed to determine whether our derivatives were able to primary recognize the same binding site of donepezil. Once evidence of this was gathered, dockings were carried out exploring a smaller grid box focused on the AChE binding sites.

**Figure 3:**
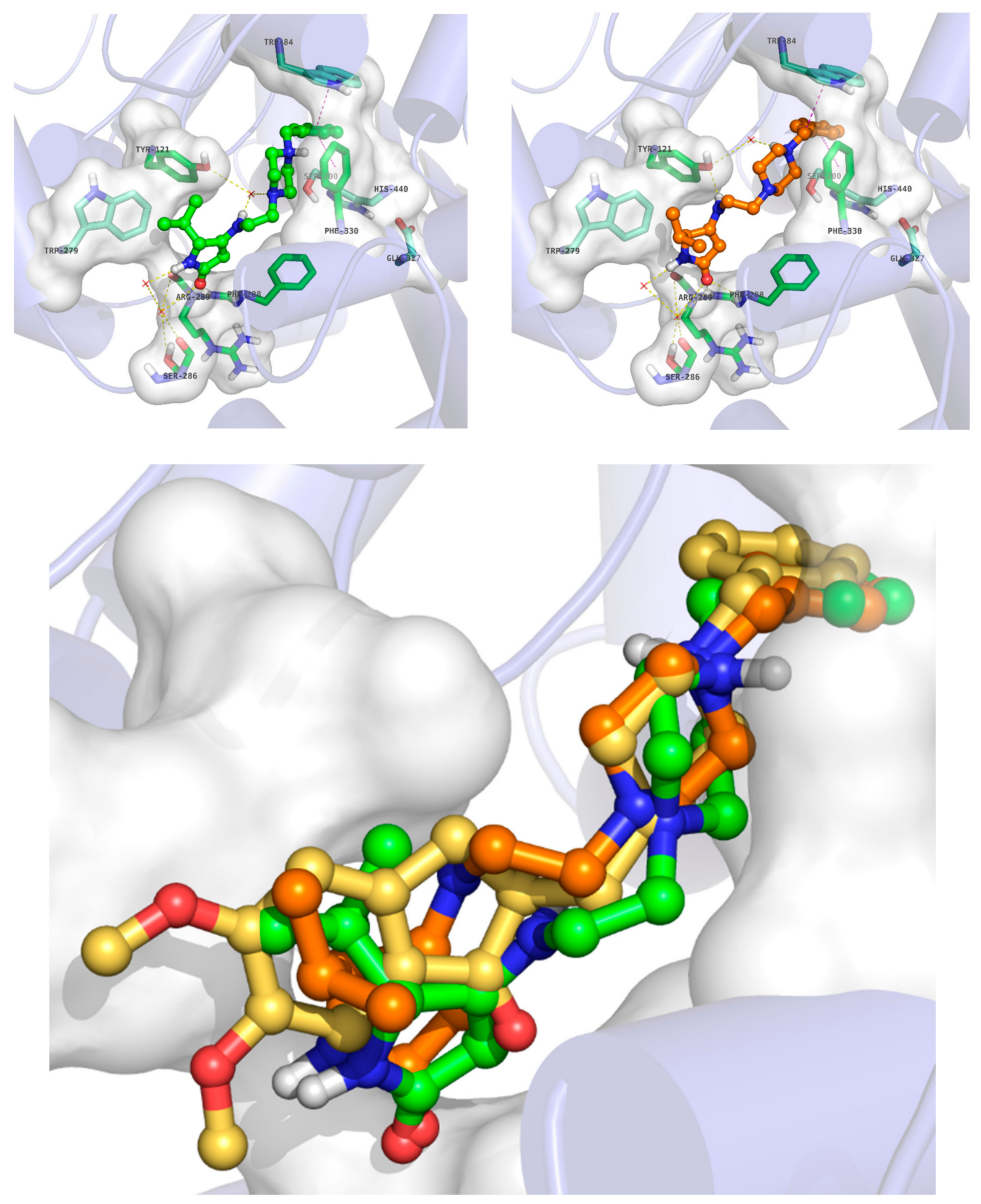
**Top**: docking poses of (*R*), (top left, green carbon atoms), and (*S*), (top right, orange carbon atoms) enantiomers of compound **5** into the eeAChE binding site (polar interactions are drawn in yellow, π − π stacking in magenta). **Bottom**: molecular superposition of the (*R*) and (*S*) with the donepezil X-ray pose (yellow carbon atoms).

In both instances, as shown in Figure 3, the benzylpiperazine moiety is well oriented and consequently accommodated at the choline binding site, facing Trp84 for an optimized face-to-face π-π interaction, largely resembling the pose of the native ligand, while the cationic piperazine ring is oriented towards the catalytic triad of CAS (Ser200, His440, Glu 327), similarly to the piperidine moiety of DNP (Figure 3, bottom). Simultaneously, the whole molecular architecture is further stabilized by a network of water mediated polar contacts involving the phenoxy group of Tyr121.

Nonetheless, the most relevant indication is perceived at the entrance of the enzyme active-site gorge, where the amide function of the five members heterocyclic ring stabilizes the binding, engaging through more than one water molecule the backbone of Ser286, Phe288 and Arg289, and the isopropyl group of the stereocenter retrieves additional hydrophobic interactions with the side chains of Trp279 and Tyr121, paralleling those found for the indanone moiety of DNP. This also guarantees good interactions with the peripheral anionic site (PAS).

It might be postulated, quite undoubtedly, that comparable interactions are gained by both the enantiomers, and as a further justification the estimated free energy of binding present very tight values gained for both configurations (−8.58 and −9.07 kcal/mol for (*R*) and (*S*), respectively).

### 2.3. Metal Complexation

Metal ions are deeply involved in the pathogenesis of neurodegenerative diseases, namely Alzheimer’s disease (AD) [34]. Cu(II) binds to Aβ peptides, thereby contributing to enable their aggregation, while deregulated redox active metal ions, Cu(II) and Fe(III), stimulate the overproduction of reactive oxygen species (ROS) with concomitant degradation of several cellular biomolecules (e.g., proteins, deoxyribonucleic acid (DNA), lipids). Therefore, one of the goals of candidate anti-AD drugs is their ability to remove deleterious ions from the affected region, restoring ionic balance without interfering with physiological ion homeostasis [35].

Thus, a model compound (**1**) containing a chelating moiety similar to that of TA was chosen to evaluate the Fe(III), Cu(II) and Zn(II) chelating ability of this series of compounds. 

#### 2.3.1. Acid-Base Properties

**Figure 4:**
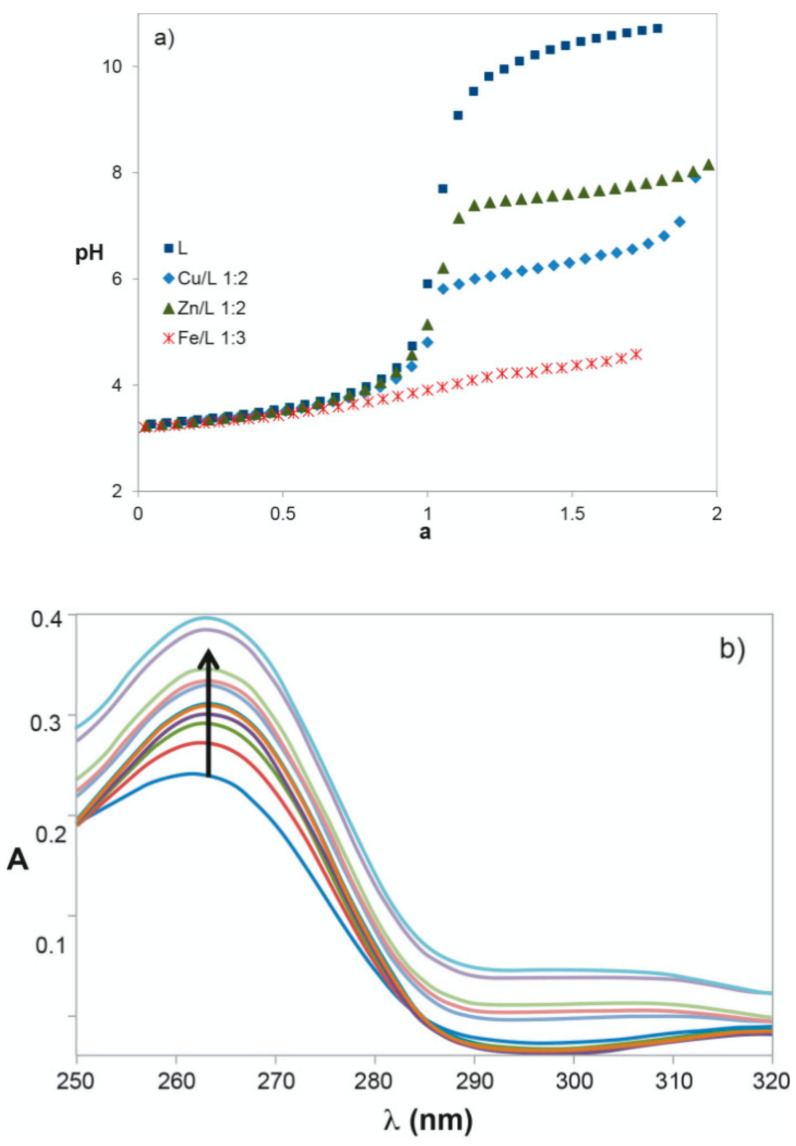
(**a**) Potentiometric titration curves of compound **1** (I = 0.1 M KCl, T = 25.0 ± 0.1 °C, C_L_ = 6.7 × 10^−4^ M) and corresponding 1:3 Fe(III)/**1** and 1:2 M((II)/**1** (M(II) = Cu(II), Zn(II)) systems in 15% *w*/*w* DMSO/water (a represents moles of added base per mole of ligand); (**b**) Spectrophotometric absorption spectra of compound **1** (2.49 < pH < 6.95) (C_L_ = 4.0 × 10^−5^ M, 15% DMSO/water) [28]. The arrow shows spectra evolution when pH increases.

In order to determine the metal complexation capacity of model compound **1**, its acid-base behavior was first studied, since it must be taken into account in the metal complexation models. A 15% *w*/*w* DMSO/H_2_O medium was adopted to perform the acid-base and chelating studies because the compound was insoluble in water. Although this medium does not seem to be compatible with biological applications, in fact, in terms of cellular studies the ligand concentration is considerably lower (<7 mM) and, therefore, the final concentration of DMSO employed in culture media would be even lower (<1%), leading to negligible alterations in biological tissues. The protonation constant of the compound was determined by pH-potentiometry and UV-Vis spectrophotometry (see Figure 4 and Table 2) by using Hyperquad 2008 [36] and Psequad [37], respectively. 

**Figure 5:**
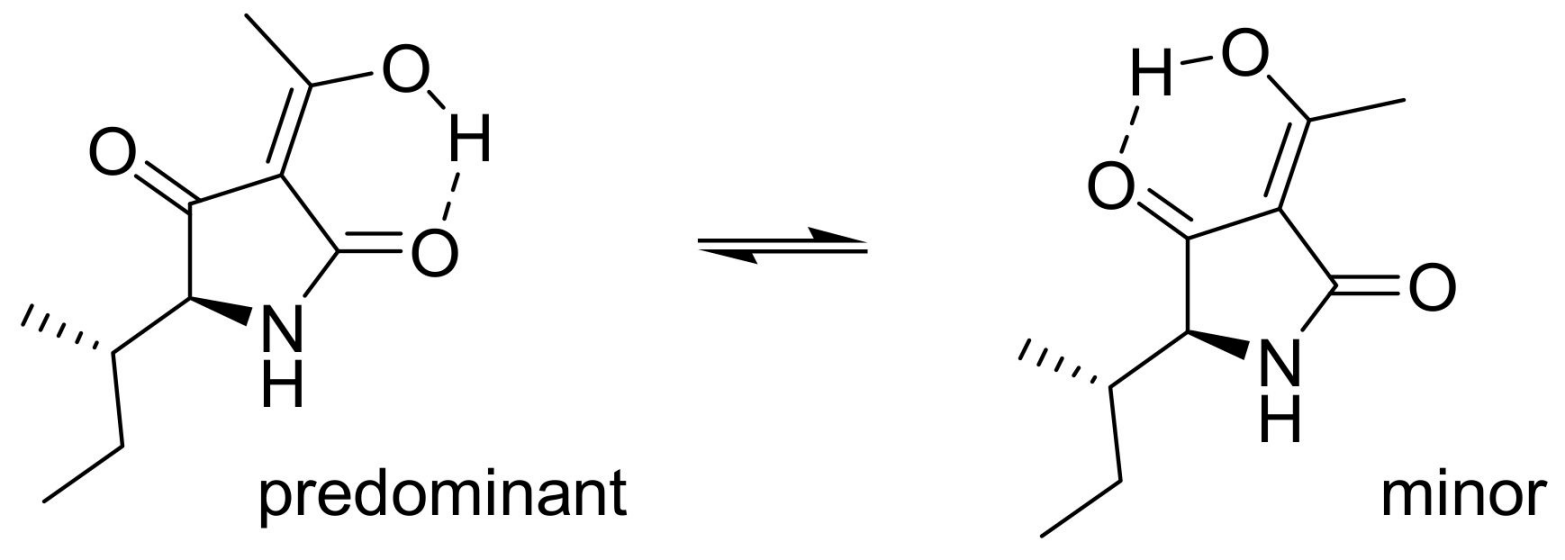
Different rotamers of TA as enolic tautomer [39].

The compound was isolated in its mono-protonated neutral form (HL in Figure 4) and it has only one dissociable proton. The corresponding protonation constant was determined both by potentiometric (log *K* = 2.334) and spectrophotometric (log *K* = 2.85) titrations (see Table 2), and the obtained values are slightly lower than the value reported in the literature for TA (log *K* = 3.523). This difference can probably be attributed to differences in the working media with different abundances of the ketonic and enolic form: compound **1** (Figure 2) has an enolic hydroxyl group directly attached to the ring, while that group is β-attached to the ring in the most abundant tautomer and rotamer of TA [39] (Figure 5). Both compounds can establish intramolecular H-bonds with 6-member-ring intermediates, but the considerable increase in the acidity of the hydroxylic proton of compound **1** suggests a stronger interaction in this case. Although it has been admitted that TA forms H-bonds under predominantly two tautomeric forms, they seem weaker than in the case of compound **1** because they involve ketone and amide groups (with known electron delocalization pushed by the amide nitrogen atom), instead of the more electron rich ester group contained in compound **1**. 

**Figure 6:**
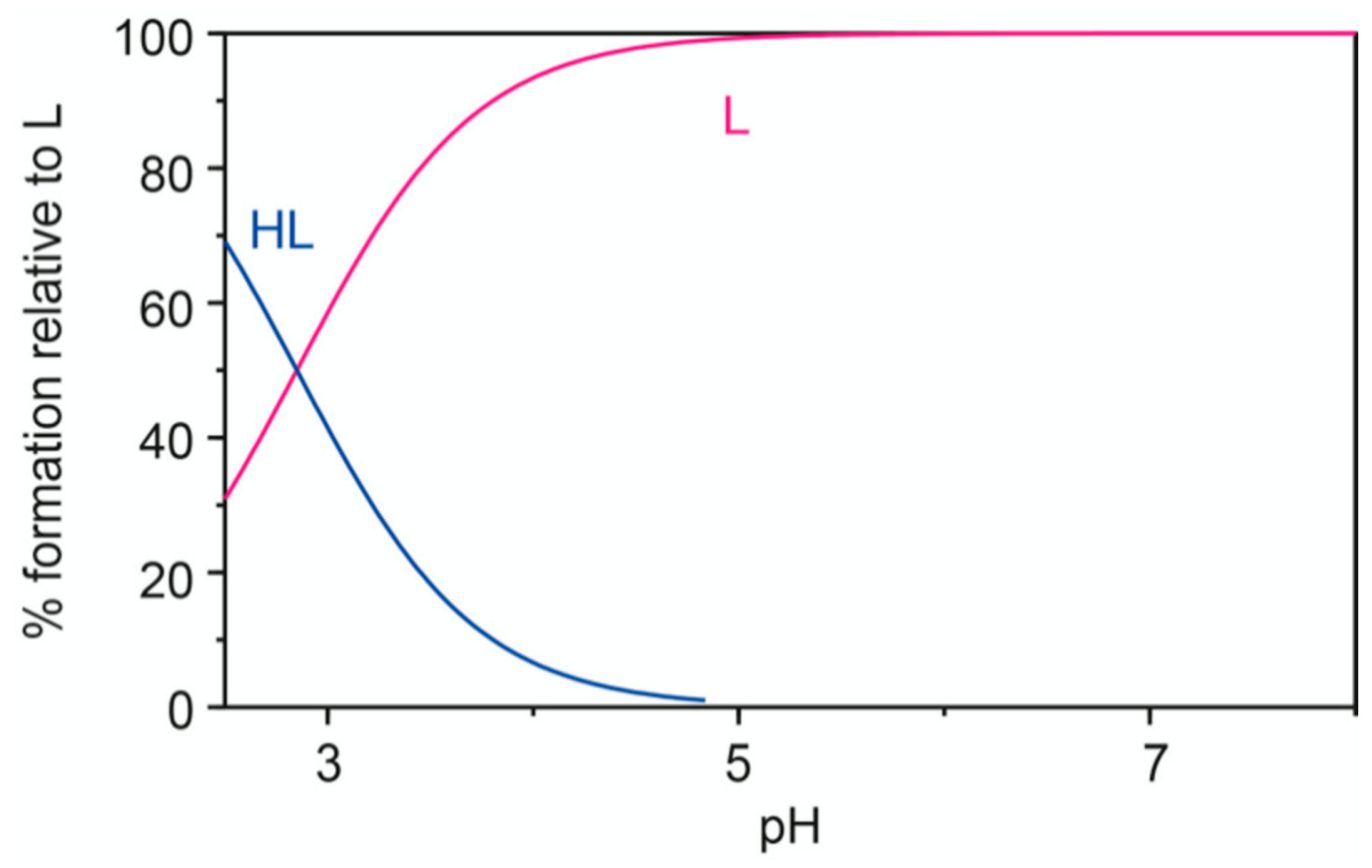
Species distribution curves for compound **1** (*C*_L_ = 4 × 10^−5^ M) in 15% *w*/*w* DMSO/water [28].

Therefore, the acidity of TA and TA derivatives **1**–**3** can be explained by the stabilizing effect resulting from the existence of different intramolecular H-bonds. Figure 6 shows the species distribution curves for compound **1** at the experimental conditions used in the spectrophotometric titration of the compound. At physiological pH 7.4, the compound is in its deprotonated negative form (L^−^).

The fact that only a low percentage of DMSO (15%) was needed in the mixed DMSO/water medium chosen to perform the solution equilibrium studies can be explained by the predominance of the mono-charged L^−^ species at physiological pH, although the lipo-hydrophilic character of a compound is determined by both the molecular charge and the ability to establish solute-solvent interactions. 

#### 2.3.2. Metal Chelation

The chelating ability of compound **1** towards Fe(III), Cu(II) and Zn(II) was evaluated through the determination of the global formation constants of the complexes via pH-potentiometric and spectrophotometric titrations of the respective 1:1, 1:2 and 1:3 M/L systems in 15% *w*/*w* DMSO/water. The fitting analysis of the respective pH-potentiometric curves was done via Hyperquad 2008 [36] while that of the spectrophotometric absorption spectra was performed using Psequad [37], and the found equilibrium models are presented in Table 2. Compound **1** was chosen because of its better profile after preliminary assays (i.e., good AChE inhibition and antioxidant activity, as well as good ability to inhibit Cu^2+-^induced Aβ aggregation). 

A significant change in the deprotonation profile of the ligand titration curve due to the presence of the metal ion was observed (see Figure 4). In fact, the curves for the M/L systems, especially for the Fe(III)/L system, lie below that of the ligand for 0 < a < 2, showing the formation of metal complexes with the deprotonated (L^−^) form of the ligand as well as mixed ligand-hydroxo metal complexes with relative stability order Fe > Cu > Zn. 

TA complexes with several metal ions, such as Fe(III), Cu(II), Ni(II) and Mg(II) were studied in detail by different techniques (microanalysis, mass spectrometry, IV spectroscopy, voltammetry) and M/L stoichiometric ratios were established: 1:2 (M = Cu, Ni, Mg) and 1:3 (M = Fe) [40]. 

The regioselectivity of metal complexation by TA has also been a matter of discussion, since two coordination sites have been pointed out: (i) between the external carbonyl (exo-enol) and the amidic carbonyl, as observed in Cu(TA)**_2_**; (ii) between the external carbonyl and the internal carbonyl (endo-enol), as observed to some degree in Fe(TA)_3_ [40,41]. 

Therefore, owing to their low log *K* value, TA and its derivatives like compound **1** are expected to be deprotonated in vivo, so compound **1** can behave as a bidentate (O,O) chelator for different metal ions, leading to six-membered coordination rings.

**Figure 7:**
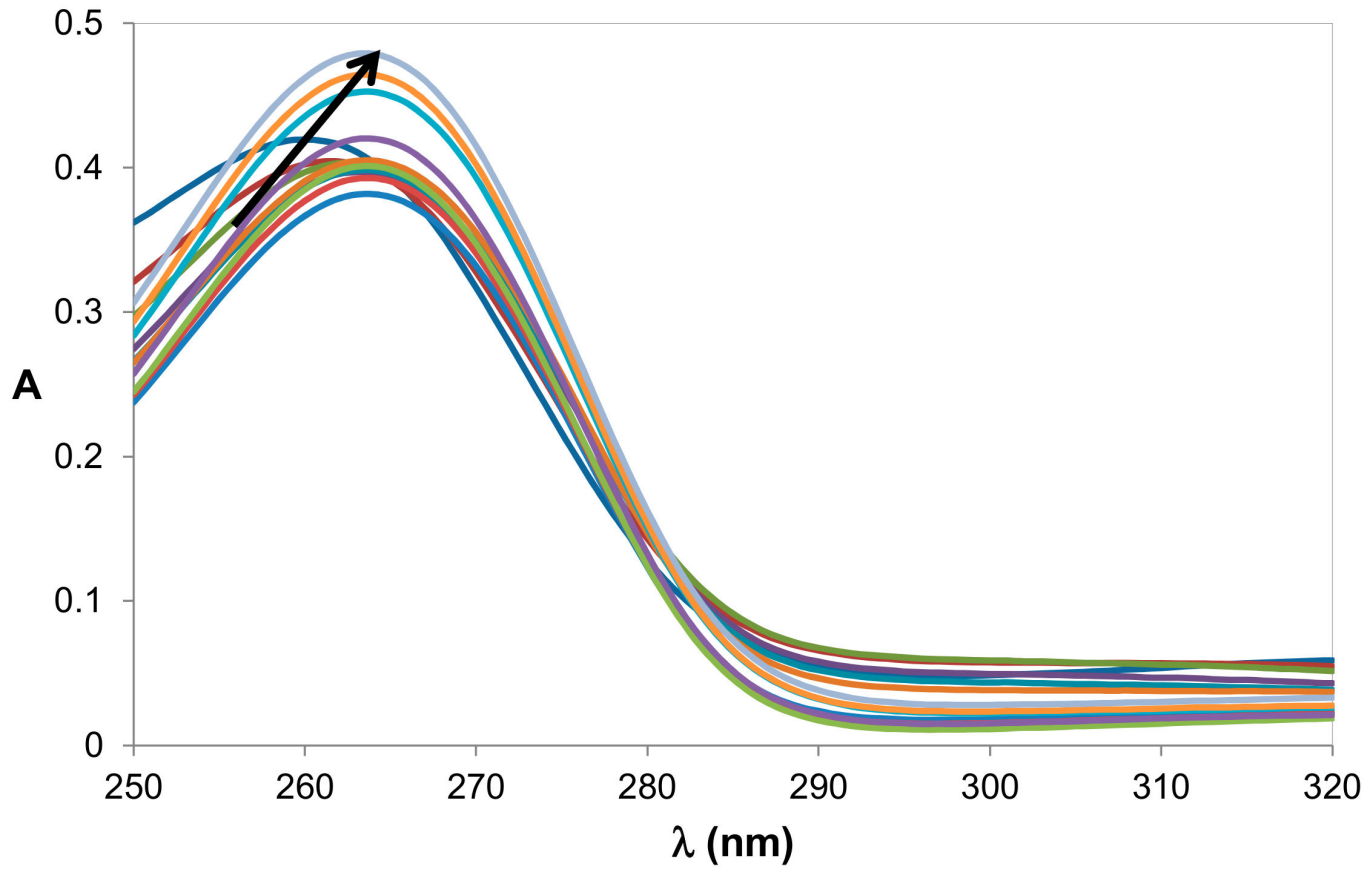
Spectrophotometric absorption spectra of the Fe^3+/^/**1** (1:1) system (2.77 < pH < 7.87) (*C*_L_ = 4.0 × 10^−5^ M, 15% DMSO/water) [28]. The arrow shows spectra evolution when pH increases.

**Figure 8:**
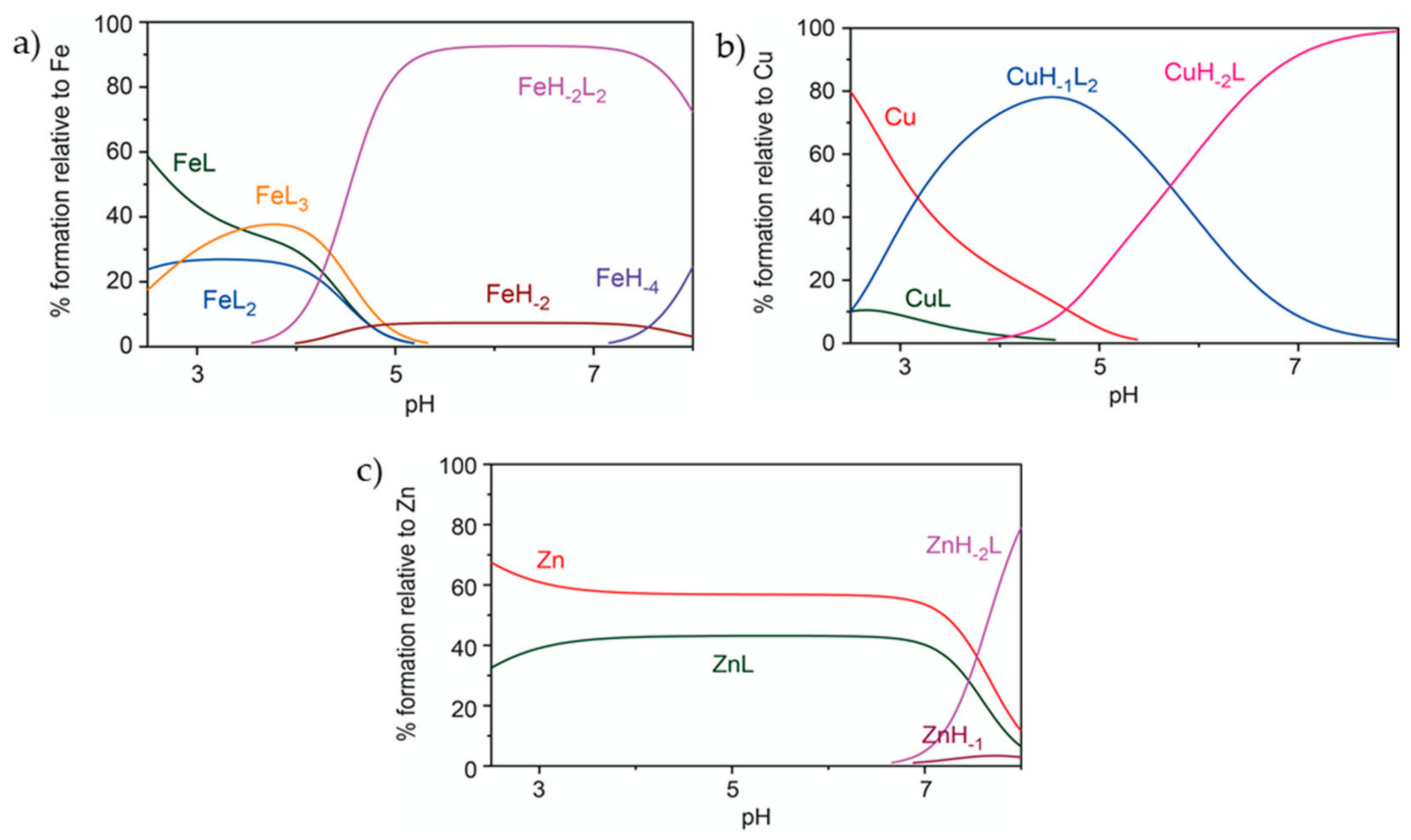
Species distribution curves for the systems: (**a**) Fe^3+^/**1** (1:3) (*C*_L_ = 4 × 10^−5^ M), (**b**) Cu^2+^/**1** (1:2) (*C*_L_ = 4 × 10^−5^ M) and (**c**) Zn^2+^/**1** (1:2) (*C*_L_ = 6.7 × 10^−4^ M) in 15% *w*/*w* DMSO/water.

To the best of our knowledge, the determination of stability formation constants of the complexes with TA has not yet been done and so the herein calculated values for compound **1** cannot be compared with those from the literature. The metal complexation equilibrium models of compound **1** were determined by the following process: for the Fe/L and Cu/L systems, only spectrophotometric data was used due to the better quality of the experimental results obtained [the 1:1 titration (e.g., Figure 7)] allowed the determination of the stability constant corresponding to the ML species and then this value, as well as the respective spectra, was introduced in the fitting of Fe/L 1:3 or Cu/L 1:2 data]; for the Zn/L system, potentiometric data was used. For the found metal complex models contained in Table 2, MH_-2_L, MH_-1_L_2_ and MH_-2_L_2_ correspond to mixed ligand-hydroxo metal complexes. Figure 8 shows the species distribution curves for the 1:3 Fe(III)/L, 1:2 Cu(II)/L and 1:2 Zn(II)/L systems at the used experimental conditions.

Figure 8 provides evidence that, at the concentrations used in the experimental conditions, complexation of Fe(III), Cu(II) and Zn(II) begins at pH below 2.5, with the formation of ML species. For the Zn(II)/L system, some competition also exists with metal hydroxide species above pH 7. Moreover, both 1:1 (CuL, CuH_-2_L, ZnL), 1:2 (FeL_2_, FeH_-2_L_2_, CuH_-1_L_2_) and 1:3 (FeL_3_) species are formed, although a 1:2 complex was not found by fitting the acquired potentiometric data for the Zn(II)/L system. The mixed hydroxo-ligand complexes (MH_-2_L, MH_-1_L_2_ and MH_-2_L_2_) are also present and denote the fact that a preferred 5-coordination core is preferential for the complex of copper with compound **1** than a square-planar geometry, while for both CuH_-2_L and ZnH_-2_L maybe a tetragonal geometry is accomplished by coordination of two water molecules.

**Figure 9:**
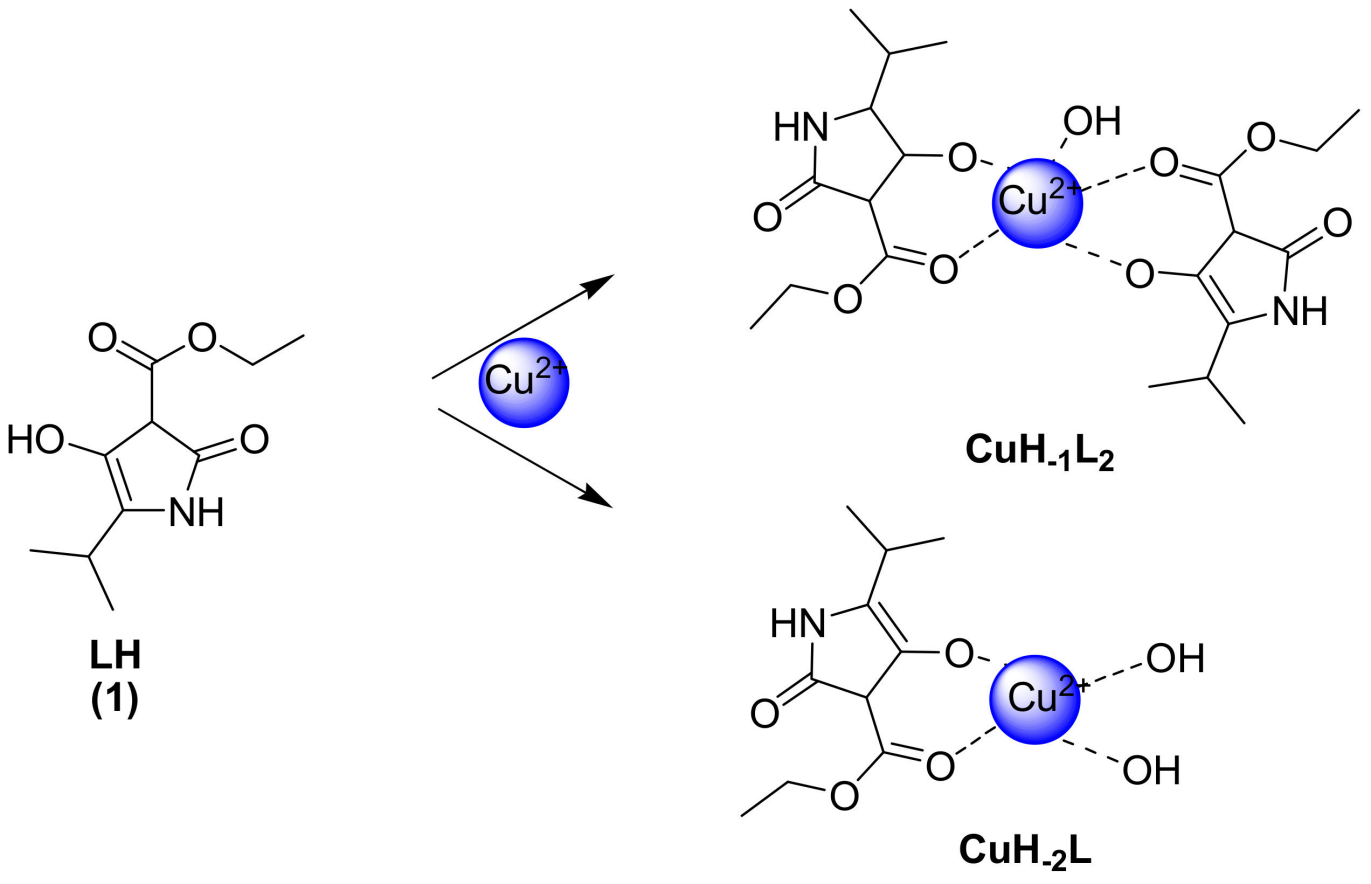
Structures of the Cu(II) complexes with compound **1** at the physiological pH [34].

Figure 9 reports possible structures for the Cu(II)/1 system at the physiological pH, evidencing the formation of 6-member chelating rings. The behavior of compounds **2** and **3**, which have an identical pharmacophore, should be the same.

Finally, it can be concluded that the TA derivatives herein studied are good iron and copper chelators (pFe(**1**) = 16.6, pCu(1) = 11.6), although only moderate for zinc (pZn (**1**) = 6.0). This stability order in metal complexation found for compound **1** is in accordance with the fact that hard bidentate (O,O) compounds prefer to coordinate to *hard* metal ions, like Fe(III), instead of *softer* ones like Cu(II) and Zn(II), following the Pearson´s principle of “hard and soft acids bases” [6]. Therefore, these TA derivatives can be considered selective iron chelators, although also good copper chelators without interfering with the biological Zn^2+^ ion.

### 2.4. Cell Viability and Neuroprotection 

**Figure 10:**
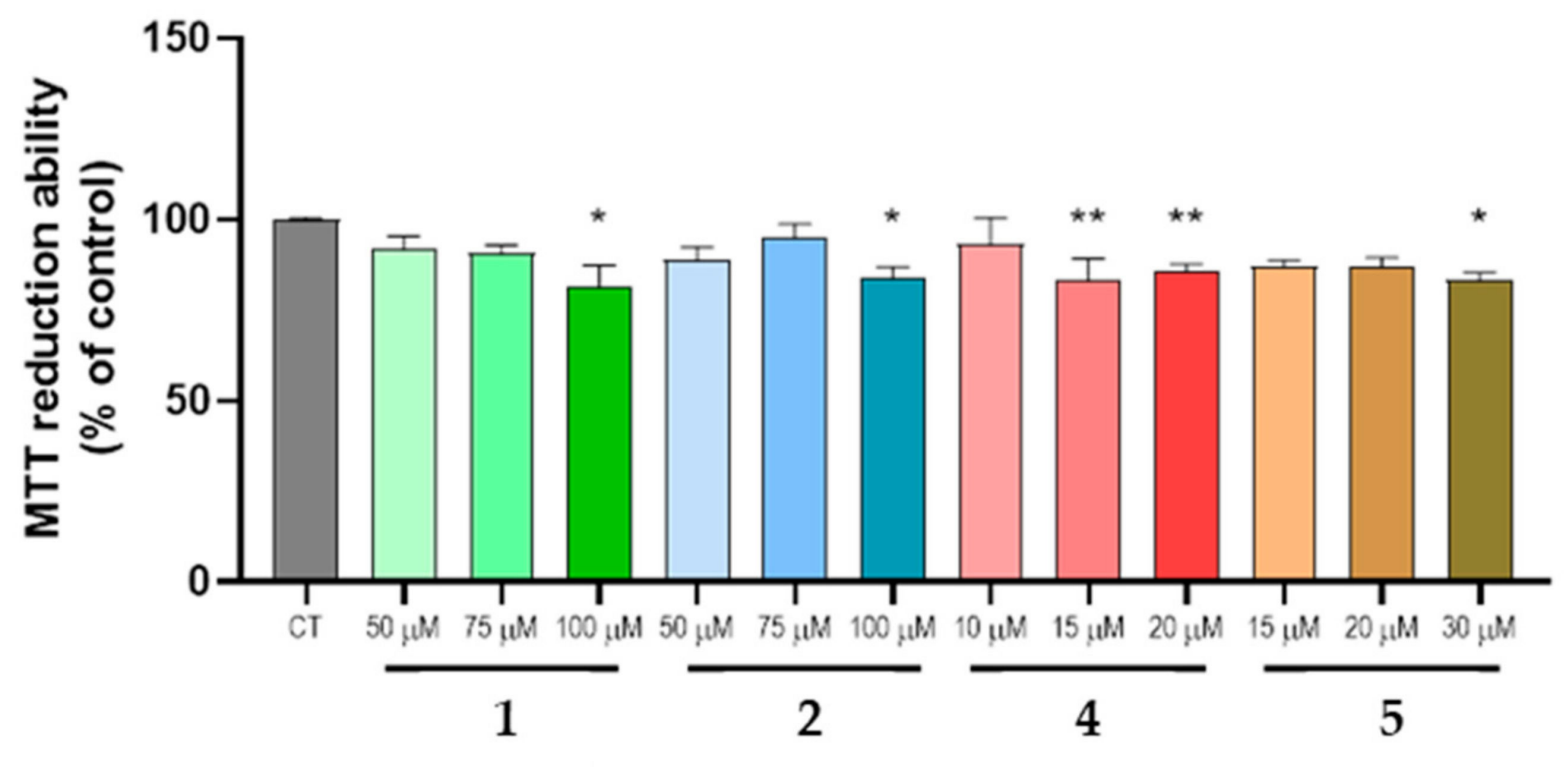
Dose-response screening of compounds **1**, **2**, **4** and **5** on SH-SY5Y neuronal cell line. Overnight cultures were treated with a range of concentrations from 10 μM to 100 μM for 24 h and cellular viability was determined by employing the MTT reduction assay. Results are expressed relatively to SH-SY5Y untreated cells, with the mean ± SEM derived from at least three different experiments. * *p* < 0.05; ** *p* < 0.01, significantly different when compared with control, untreated SH-SY5Y cells.

Since an effective drug targeting Aβ oligomerization is yet to be found, the emergence of new disease-modifying agents is crucial for the development of new therapies. With this in mind, we performed a dose-response screening to determine the highest non-cytotoxic concentration of selected therapeutic candidates, namely **1** and **2**, and their respective derivatives **5** and **4** (Figure 10). This assay was also useful to demonstrate that, at the active concentrations, these molecules do not show (except for compound **4**) cytotoxicity on the tested cell line.

**Figure 11:**
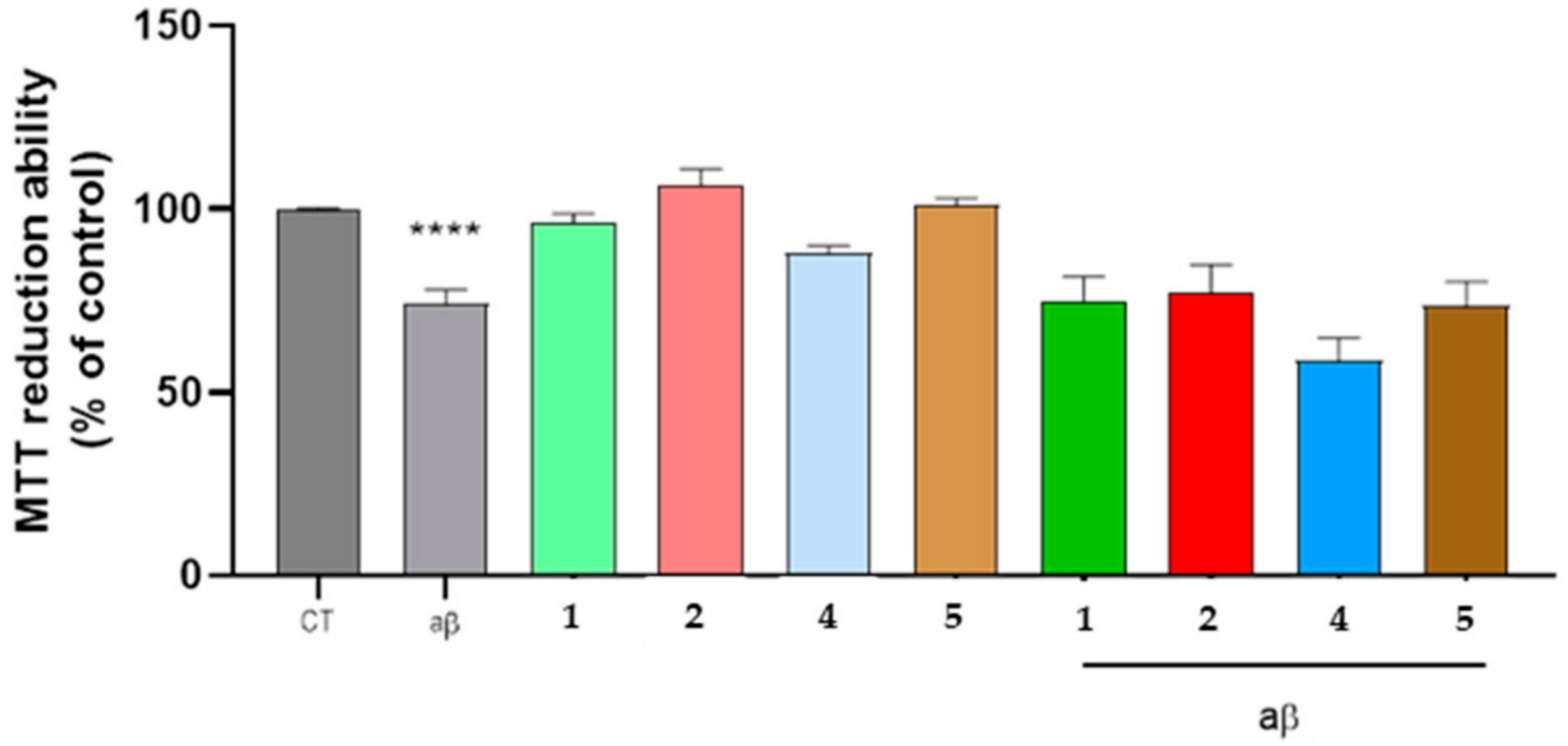
Selected compounds do not confer protection against Aβ_1-42_ -induced toxicity on SH-SY5Y cells. Overnight cultures were treated with Aβ_1-42_ peptide (2.5 μM) for 24 h in the absence or in the presence of the compounds. When compounds were present, a pre-incubation of 1 h preceded the Aβ_1-42_ treatment. Cellular viability was determined by employing the MTT reduction assay. Results are expressed relatively to SH-SY5Y untreated cells, with the mean ± SEM derived from at least three different experiments. **** *p* < 0.0001, significantly different when compared with SH-SY5Y untreated cells. (compounds: **1**, **2**–100 µM; **4**–10 µM; **5**–20 µM).

At the molecular level, this multifactorial disease is characterized by the intracellular formation of neurofibrillary tangles and by a marked extracellular deposition of senile plaques. These plaques are fibrillar depositions mainly composed of Aβ_1-42_ peptides, which in turn oligomerize into fibrils and are then externalized by neurons. Hence, the administration of Aβ_1-42_ peptides to SH-SY5Y cells is a useful cellular model to investigate the pathophysiology of AD. Indeed, administration of Aβ_1-42_ was sufficient to induce cell toxicity in cells (Figure 11). Our results demonstrate that the tested compounds did not directly protect from Aβ_1-42_-induced toxicity (Figure 11).

**Figure 12:**
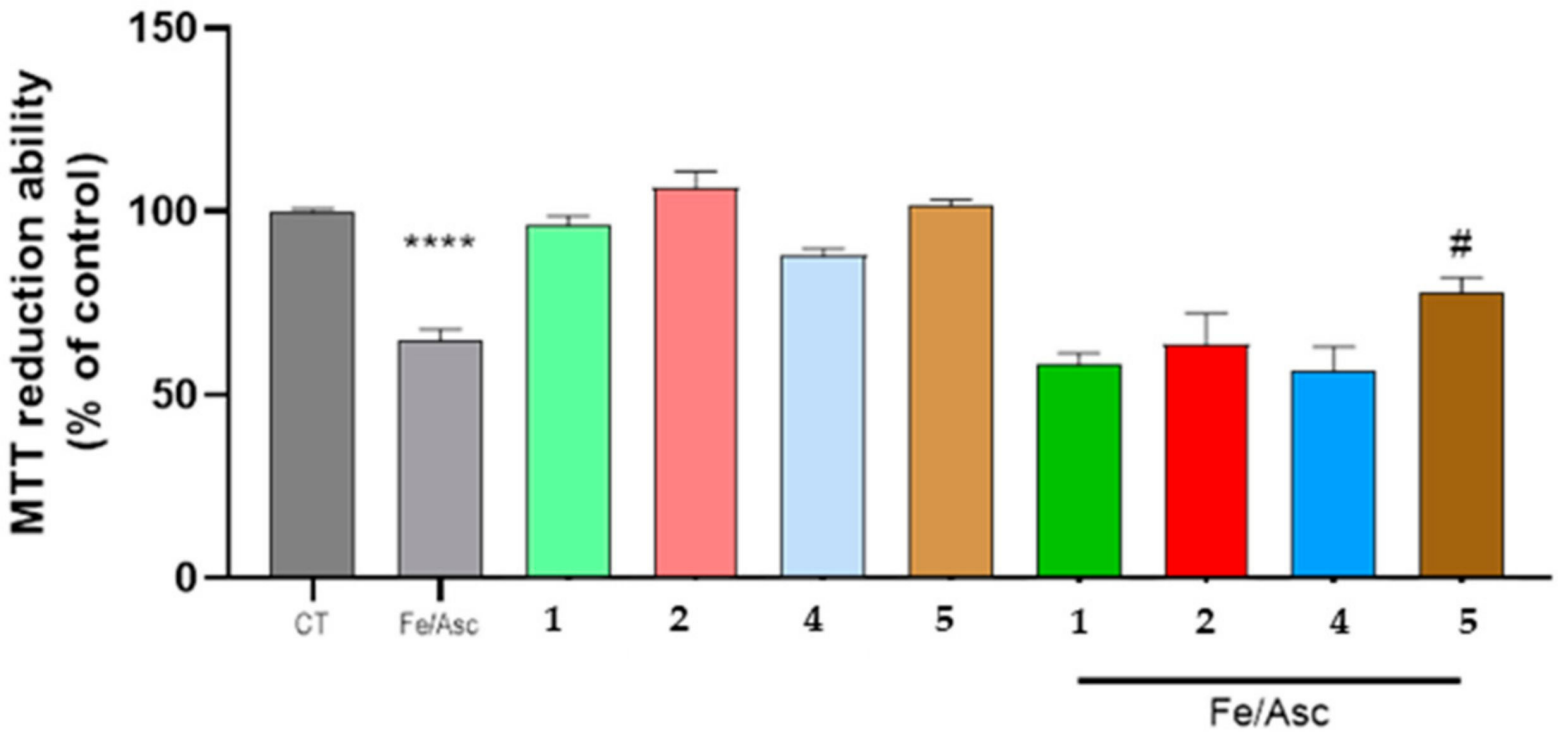
Neuroprotective effect of compounds after Ferrous Sulfate (Fe)/L-Ascorbic Acid (AscH(-)) insult. SH-SY5Y cells were treated with Fe/Asc (2.5 M/5 mM, respectively) for 24 h in the absence or in the presence of compounds. When compounds were present, a pre-incubation of 1 h preceded the Fe/Asc treatment. Cellular viability was determined by employing the MTT reduction assay. Results are expressed relatively to SH-SY5Y untreated cells, with the mean ± SEM derived from at least three different experiments. **** *p* < 0.0001, significantly different when compared with SH-SY5Y untreated cells; # *p* < 0.05, significantly different when compared with Fe/Asc treated SH-SY5Y cells. (compounds: **1**, **2**–100 µM; **4**–10 µM; **5**–20 µM).

However, it must be emphasized that oxidative stress derived from intense production of reactive oxygen species is a another important hallmark of AD [42]. Interestingly, Aβ plaques deposition is preceded by an increase in ROS production [43]. In fact, mitochondrial disruption leads to an increase in ROS production, leading to an oxidative-stress mediated deposition of amyloid plaques [44]. Additionally, oxidative stress has a meaningful impact in AD, since the levels of oxidized proteins and DNA are increased in the brain of AD patients [45]. As such, we sought to utilize the combination of Fe/Asc to induce oxidative stress in our cellular model, in order to study a possible positive effect of selected compounds in the early stages of the pathology. As observed in Figure 11, Fe/Asc was able to arrest SH-SY5Y proliferation. In our assay, compound **5** presented the best capacity to scavenge oxygen radicals and conferred neuroprotection against the Fe/Asc challenge (Figure 12). This is preferable because drugs that target enzymes involved in the redox state regulation are a better option than radical scavengers in antioxidant therapy. Since compound **5** was not able to directly protect SH-SY5Y cells from Aβ_1-42_-induced cell toxicity, we believe that it can be beneficial in the earlier stages of AD by ameliorating the oxidative status of cells.

### 2.5. Pharmacokinetic Properties

Pharmacokinetic studies were performed using QikProp v.2.5. [46], in an effort to assess the drug-likeness of the novel compounds and their potential to penetrate important membranes such as the blood–brain barrier (BBB). Moreover, analysis of parameters such as calculated log P (clog *P*) can be useful to address the design of new compounds. From the analysis of the results reported in Table 3, it is evident that all the compounds do not show violations of “Lipinski’s rule of five” [47] and this indicates a potential good oral bioavailability. The results calculated for the BBB permeability (log BB), and the octanol-water partition coefficient (clog *P*, are within the range of values calculated for known drugs, respectively, (−3–1.2) and (−2–6.5) [46]. Furthermore, the small sized compounds **1**–**3** present good CACO-2 permeability (absorption from the intestinal tract to the blood) but low CNS activity. These are still encouraging results, in view of expected further structural derivatizations. On the other hand, compounds **4** and **5**, presenting a high CNS activity, can be considered potentially effective as final anti-AD drug candidates.

**Figure 13:**
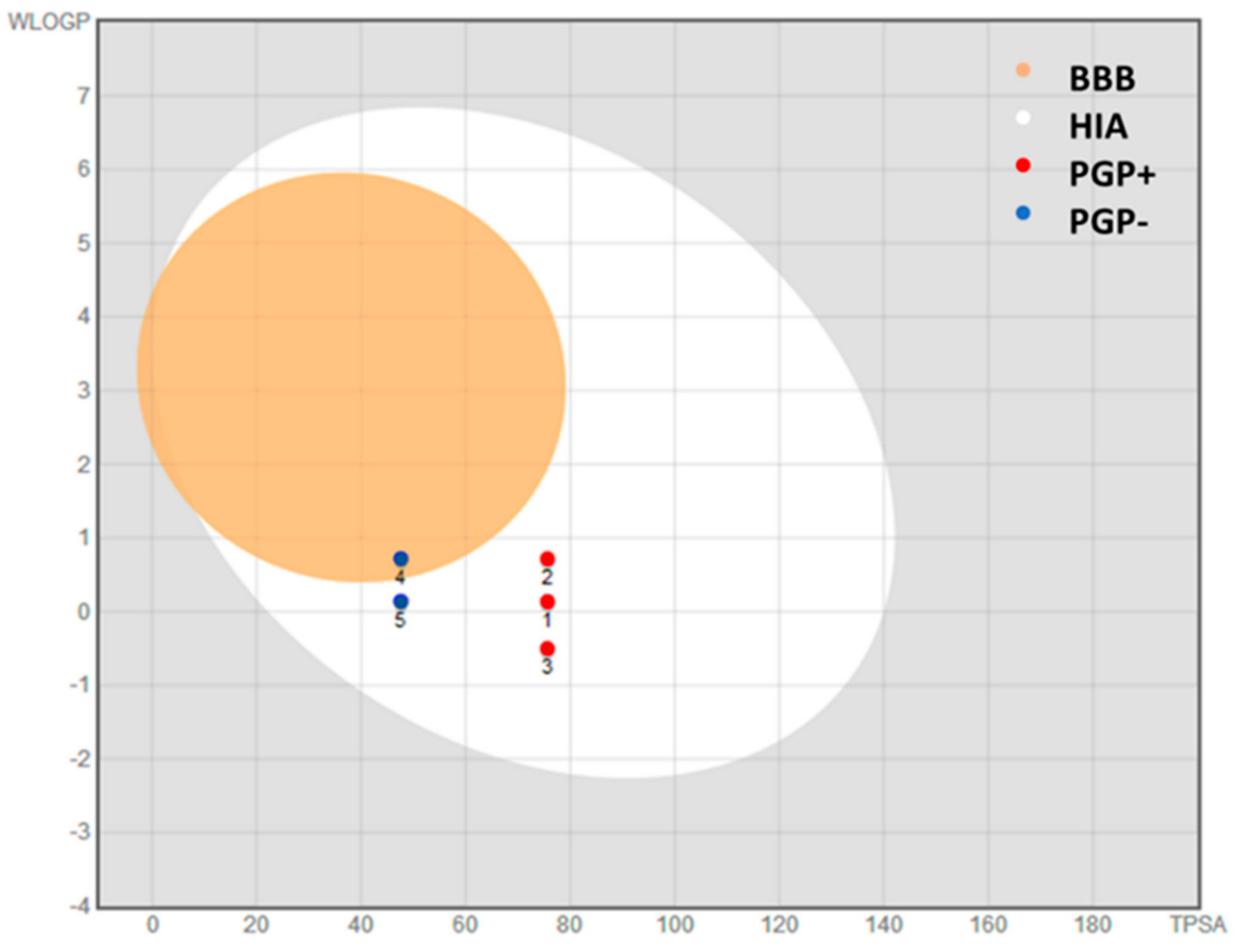
Pharmacokinetic profile of compounds **1**–**5** according to BoiledEgg. BBB = blood-brain barrier permeation; HIA = human intestinal absorption; PGP+ = potential substrate of P-glycoprotein, PGP− = non potential substrate of P-glycoprotein.

Moreover, this assumption was corroborated further thanks to drug-likeness predictions made with the Brain Or IntestinaL EstimateD permeation method [48], which suggested that all studied compounds are well absorbed in the gastrointestinal tract due to their lipophilicity and polarity (measured by WLOGP and TPSA, respectively), shown in the white ellipse of the BOILED-Egg plot (see Figure 13). This graph confirms that compound **4** (and probably **5** as well) might be able to pass the blood-brain barrier and could therefore be active in the Central Nervous System. Consequently, these molecules can be definitely considered as promising hit compounds in view of a future structure–activity relationship study.

## 3. Materials and Methods 

### 3.1. Chemicals

Chemicals were purchased from Merck Life Science (Milan, Italy) and were utilized without any further purification. The reactions were monitored via thin-layer chromatography (TLC; silica gel, F254) with UV light (short-wave ultraviolet 254 and 365 nm). Inert atmosphere of nitrogen was used to carry out all reactions that required an anhydrous environment. Column chromatography was conducted using 60 Å silica gel (63–200 μm, Fluka, Milan, Italy). Mass spectrometry was conducted on a HP MS 6890-5973 MDS spectrometer, electron impact 70 eV, equipped with a HP ChemStation or with an Agilent 6530 Series Accurate-Mass Quadrupole Time-of-FLIGHT (Q-TOF) LC/MS (Agilent, Santa Clara CA, USA). A Bruker (Billerica, MA, USA) micro-TOF QII mass spectrometer equipped with an electrospray (ESI) ion source was used to carry out HRMS (high-resolution mass spectroscopy) analyses. ^1^H NMR spectra were obtained deuterated solvents on a Varian Mercury 300 (Varian Inc., Palo Alto, CA, USA) and an Agilent VNMRS500 spectrometer (Agilent, Santa Clara CA, USA). Chemical shifts (δ) are reported as parts per million (ppm) and coupling constants (J) in Hertz (Hz). Melting points are uncorrected and were measured on a Gallenkamp electrothermal apparatus (Fisons Erba Science Ltd., Guildford, UK) in open capillaries.

### 3.2. Synthesis of Chemical Compounds

#### 3.2.1. Synthesis of 2-(2-4-Benzylpiperazinil-1-yl)ethyl)isoindoline-1,3-dione

Phthalic anhydride (10 mmol) and 1-(2-aminoethyl)-piperazine (10 mmol) were heated at 160 °C for 4 h [13]. The resulting dark brown solid was dissolved in 96° ethanol (25 mL) and added with KOH (11 mmol) and benzyl bromide (10 mmol). The mixture was stirred at room temperature (RT) for 24 h. Then, the ethanol was removed in vacuo and the residue was partitioned between diethyl ether and H_2_O. The aqueous layer was further extracted with diethyl ether (3 times), and the collected organic phases were washed with brine, dried over anhydrous Na_2_SO_4_, filtered and concentrated under reduced pressure obtaining a pale yellow solid. Yield: 53%. ^1^H-NMR (400 MHz, CDCl_3_), δ (ppm): 2.39–2.52 (m, 8H, piperazine); 2.60 (t, 2H, *J* = 6.7 Hz, phthalimide CH_2_CH_2_N); 3.44 (s, 2H, NCH_2_Ph); 3.78 (t, 2H, *J* = 6.7 Hz, phthalimide CH_2_CH_2_N); 7.19–7.27 (m, 5H, aromatics, PhCH2); 7.67–7.69 and 7.80–7.82 (m, 4H, phthalimide). GC-MS *m*/*z* (%): 349 (7) [M]^+^, 189 (100), 91 (47). HRMS [M + H]^+^: calculated 350.1863; found 350.1858.

#### 3.2.2. Synthesis of 2-(4-Benzyl-1-piperazinyl)ethanamine (6)

2-(2-(4-Benzylpiperazin-1-yl)ethyl)isoindoline-1,3-dione (1 mmol) was dissolved in an aqueous solution of MeNH_2_ 40% (*w*/*w*, 5 mL). Then, the mixture was stirred at room temperature for 60 h. Subsequently, an aq. solution of 20% (*w*/*w*) NaOH (5 mL) was added and the resulting mixture was stirred for 2 h. Then, NaCl (1 mmol) was added and the solution was extracted with dichloromethane and the organic layer washed with water, dried over anhydrous Na_2_SO_4_, filtered and concentrated under reduced pressure affording a pale-yellow oil. Yield: 85%. ^1^H-NMR (400 MHz, CDCl_3_), δ (ppm): 2.35–2.43 (m, 10H, piperazine, NCH_2_CH_2_N); 2.73 (t, 2H, *J* = 6.1 Hz, NCH_2_CH_2_N); 3.46 (s, 2H, NCH_2_Ph); 7.18–7.27 (m, 5H, aromatics, PhC). GC-MS *m*/*z* (%): 219 (1) [M]^+^, 189 (100), 91 (94). HRMS [M + H]^+^: calculated 220.1808; found 220.1809.

#### 3.2.3. General Procedure for the Synthesis of Compounds **1a**–**3a**

Ethyl malonyl chloride (5 mmol), was added at 0 °C to a solution of a commercial aminoacid (alanine, or phenylalanine, or valine) methyl ester (6 mmol) and triethylamine (TEA, 11.5 mmol) in anhydrous CH_2_Cl_2_ and the reaction mixture was stirred for 24 h, under nitrogen atmosphere at room temperature. Then, the solution was washed with NaHCO_3_ and brine solutions, dried over anhydrous Na_2_SO_4_, filtered, and concentrated under reduced pressure, affording a yellow oil.

*Methyl 2-((3-ethoxy-3-oxopropanamido)methyl)-3-methyl-butanoate* (**1a**). Obtained starting from valine methyl ester hydrochloride. Yield: 95%. ^1^H-NMR (300 MHz, CDCl_3_), δ (ppm): 0.91 (t, 6H, CH_3_CHCH_3_); 1.28 (t, 3H, OCCH_3_); 1.88-1.95 (m, 1H, CH); 3.33 (s, 3H, OCH_3_); 3.72 (s, 2H, COCH_2_CO); 4.21 (q, 2H, OCH_2_C); 4.58 (dd, 1H, NHCH); 7.59 (d, 1H, NH). GC-MS *m*/*z* (%): 245 (1) [M]^+^; 186 (100); 88 (25); 72 (42).

*Ethyl 3-((2-benzyl-3-methoxy-3-oxopropyl)amino)-3-oxopro-panoate* (**2a**). Obtained starting from phenylalanine methyl ester hydrochloride. Yield: 90%. ^1^H-NMR (300 MHz, CDCl_3_), δ (ppm): 1.26 (t, 3H, OCCH_3_); 3.09 (dd, 1H, PhCCH) and 3.17 (dd, 1H, PhCH_2_C); 3.72 (s, 3H, OCH_3_); 4.18 (q, 2H, OCH_2_); 4.87 (m, 1H), 7.12–7.29 (m, 5H, aromatics) and 7.44 (1H, d, NH). GC-MS *m*/*z* (%): 293 (1) [M]^+^; 162 (100); 88 (48). 

*Ethyl 3-((3-methoxy-2-methyl-3-oxopropyl)amino)-3-oxopro-panoate* (**3a**). Obtained starting from alanine methyl ester hydrochloride. Yield: 71%. ^1^H-NMR (300 MHz, CDCl_3_), δ (ppm): 1.28 (t, 3H, CCH_2_); 1.42 (d, 3H, CH_3_C); 3.32 (s, 2H, COCH_2_CO); 3.74 (s, 3H, OCH_3_); 4.20 (q, 2H, OCH_2_C); 4.59 (m, 1H); 7.61 (s, 1H, NH). GC-MS *m*/*z* (%): 217 (1) [M]^+^; 158 (100); 44 (79).

#### 3.2.4. General Procedure for the Synthesis of Compounds **1**–**3**

Sodium ethoxide was prepared dissolving Na (4 mmol) in absolute ethanol (9 mL). Then the suitable substrate (**1a**–**3a**, 1 mmol) was added and the resulting mixture was stirred and refluxed for 4 h. The absolute ethanol was removed under reduced pressure and the crude was diluted with distilled water and acidified with 2N HCl to pH 2 and extracted with CH_2_Cl_2_. The collected organic phases were then washed with brine, dried over anhydrous Na_2_SO_4_ and concentrated under reduced pressure to obtain the title compounds as orange solids. 

*Ethyl 4-hydroxy-5-isopropyl-2-oxo-2,5-dihydro-1H-pyrrole-3-carboxylate* (**1**). Yield: 51%. m.p. = 100–103 °C. ^1^H-NMR (300 MHz, CDCl_3_), δ (ppm): 0.89 and 1.05 (2 d, 3H + 3H, CH_3_CCH_3_); 1.39 (t, 3H, CH_3_C); 2.17–2.24 (m, 1H, CCHC); 4.08 (d, 1H, NHCH); 4.36–4.44 (q, 2H, CCH_2_). ESI-MS *m*/*z*: 214 [M + 1]^+^. HRMS (C_10_H_15_NO_4_+Na^+^): calculated: 236.0893 found: 236.0893. 

*Ethyl 5-benzyl-4-hydroxy-2-oxo-2,5-dihydro-1H-pyrrole-3-carboxylate* (**2**). Yield: 79%. m.p. = 105–107 °C. ^1^H-NMR (300 MHz, CD_3_OD), δ (ppm): 1.39 (t, 3H, CH_3_C); 2.70 (dd, 1H, PhCCH); 3.28 (dd, 1H, PhCCH); 4.33-4.43 (m, 2H, CCH_2_ + 1H CH); 5.63 (s, 1H, OH); 7.19–7.32 (m, 5H, aromatics). ESI-MS *m*/*z*: 260 [M − 1]^−^. HRMS (C_14_H_15_NO_4_+Na^+^): calculated: 284.0893 found: 284.0894. 

*Ethyl 4-hydroxy-5-methyl-2-oxo-2,5-dihydro-1H-pyrrole-3-carboxylate* (**3**). Yield: 41%. m.p. = 103–105 °C. ^1^H-NMR (300 MHz, CD_3_OD), δ (ppm): 1.37–1.46 (m, 6H, 2 CH_3_); 4.21 (q, 1H, CHC); 4.42 (q, 2H, CCH_2_); 5.88 (s, 1H, OH). ESI-MS *m*/*z*: 186 [M + 1]^+^ 208 [M + Na]^+^. HRMS (C_8_H_11_NO_4_+Na^+^): calculated: 208.0580 found: 208.0571.

#### 3.2.5. General Procedure for the Synthesis of Compounds **4** and **5**

Intermediate 6 was added to a solution of compound **2** or **1** in THF [23]. After 5 h reflux, the solution was dried to evaporate the THF affording a brown oil. Then, the crude was purified affording the title compounds 4 and 5 as white solids. The compound **4** (R = CH_2_Ph) was purified through crystallization with *n*-hexane and chloroform. The compound **5** (R = isopropyl) was purified through chromatography column (CH_2_Cl_2_:MeOH:TEA 98:2:0.1) and then crystallization with ethyl acetate and chloroform to obtain a white solid. 

*5-Benzyl-4-((2-(4-benzylpiperazin-1-yl)ethyl)amino)-1,5-di-hydro-2H-pyrrol-2-one* (**4**). Crystallized from *n*-hexane and chloroform. Yield: 26%. m.p. = 173–174 °C. ^1^H-NMR (300 MHz, CD_3_OD), δ (ppm): 2.47–2.59 (m, 8H piperazine + 2H CH_2_piperazine); 2.76 (dd, 2H, CHCPh); 3.00 (dd, 2H, CH_2_ piperazine); 3.08 (m, 2H, CH_2_); 3.53 (s, NCH_2_Ph); 4.21 (q, 1H, CH); 4.60 (s, 1H); 4.97 (s, 1H, NH); 5.17 (s, 1H, NH); 7.22–7.31 (m, 10H, aromatics). ESI-MS *m*/*z* (%): 413 [M + Na]^+^, HRMS (C_24_H_30_NO_4_+Na^+^): calculated: 413.2312 found: 413.2317.

*4-((2-(4-Benzylpiperazin-1-yl)ethyl)amino)-5-isopropyl-1,5-dihydro-2H-pyrrol-2-one* (**5**). Purified through chromatography column (CH_2_Cl_2_:MeOH:TEA 98:2:0.1) and then crystallization with acetate and chloroform. Yield: 25%. m.p. = 168–170 °C. ^1^H-NMR (300 MHz, CD_3_OD), δ (ppm): 0.77 and 1.05 (2 d, 3H + 3H, CH_3_CCH_3_); 1.90 (m, 1H, CCHC); 2.49–2.65 (m, 11H, 8H piperazine + 2H CCH_2_Ph + 1H CH); 3.10 (m, 2H CH_2_); 3.52 (s, 2H, NCH_2_Ph); 4.65 (s, 1H, CH); 4.96 (s, 1H, NH); 5.17 (s, 1H, NH); 7.26–7.32 (m, 5H, aromatics). GC-MS *m*/*z* (%): 342 (1) [M]^+^; 189 (100) [C_12_H_17_N_2_]^+^; 91 (33) [C_7_H_7_]^+^. ESI-MS *m*/*z* (%): 365 [M + Na]^+^, 343 [M + 1]^+^, 341 [M − 1]^−^. HRMS (C_20_H_30_NO_4_-H^−^): calculated: 341.2341 found: 341.2354.

### 3.3. Bioanalytical Procedures

#### 3.3.1. Acetylcholinesterase (AChE) Inhibition

Enzyme inhibition assays were performed using an adaptation of Ellman’s method [30]. The readings were made in a spectrophotometer Perkin-Elmer Lambda 35 UV-Vis. Enzyme activity is measured by following the increase in yellow color produced from thiocholine when it reacts with dithiobisnitrobenzoate ion (DTNB). DTNB solution was obtained dissolving the reagent in HEPES buffer with salts, namely NaCl (50 mM) and MgCl_2_ (20 mM), obtaining a 3 mM solution. The enzyme AChE of electroforus electricus (electric eel) stock (500 U) was dissolved in TRIS 50 mM, pH 8.0, being posteriorly diluted to assay concentrations (5 U), obtaining an aliquot of 1 mL. The enzyme substrate used was acetylthiocholine iodide (AThCh, 16 mM), which was prepared with deionized water. The compounds tested were diluted in MeOH or DMSO (1 mg/mL, 2–7 × 10^−3^ M), with further dilution, and five different concentrations were used for each compound, obtaining different percentages of AChE inhibition. The assay consists in the addition of HEPES (50 mM, pH = 8), inhibitor, AChE and MeOH in a 1 mL cuvette. The mixture was left to incubate for 15 min and, after that, AThCh and DTNB were added. The enzymatic reaction was followed through the measurements of absorbencies at 405 nm (Perkin Elmer Lambda 35 spectrophotometer equipped with a temperature programmer PTP 1þ1 Peltier System, using thermostated (T = 25.0 ± 0.1 °C) 1 cm path length cells) during 5 min. A blank solution containing all the components except the enzyme, that was substituted for equal volume of HEPES, was used. A control reaction was used using MeOH instead of the new compounds and was considered the reaction corresponding to 100% enzymatic activity. Each assay was performed in quintupled [12,13,14]. 

#### 3.3.2. Radical Scavenging Capacity

The DPPH (2,2-diphenyl-1-picrylhydrazyl) method was used to determine radical scavenging activity consisting in the addition of different volumes of stock solution of compound (4 × 10^−4^ − 6 × 10^−3^ M in MeOH) to a methanolic solution of 2.5 mL of DPPH [13]. The final volume of the assay was 3.5 mL, corrected with MeOH to obtain different concentrations of our compound in each assay. The control solution was composed of MeOH and DPPH [14]. After 30 min, at RT, protected from light, the absorbance was measured at 517 nm (Perkin Elmer Lambda 35 spectrophotometer equipped with a temperature programmer PTP 1þ1 Peltier System, using thermostated (T¼25.0 ± 0.1 °C) 1 cm path length cells), against a blank solution (MeOH). The assays were performed in triplicate, in which the EC_50_ was obtained through the graphical representation of the %AA vs. the inhibitor concentration. 

#### 3.3.3. Inhibition of Aβ Aggregation

The spectrofluorimetric method, based on the fluorescence emission of thioflavin T (ThT), was used to study the Aβ_1-42_ aggregation inhibition. Firstly, Aβ_1-42_ was dissolved in HFIP (1,1,1,3,3,3-Hexafluoro-2-propanol) and kept overnight at room temperature [12,13,14] in order to obtain a dry film. Then, the film was re-dissolved in CH_3_CN-Na_2_CO_3_ (300μM)/NaOH (250μM) (48.3/48.3/3.4 mL *v*/*v*/*v*) and then phosphate buffer (pH = 8) was added. The ligand solutions were prepared with MeOH (1mg/mL) and further with phosphate buffer. For the inhibition studies of copper-induced Aβ aggregation, a solution of CuCl_2_ 240 μM was used. As a control, a solution of 30 μL of phosphate buffer and 30 μL of Aβ_1-42_ was used to observe the aggregation without any inhibition, while the ligand assay solutions were composed of 20 μL of phosphate buffer, 30 μL of Aβ_1-42_ and 10 μL of ligand. The Cu-induced aggregation studies were similarly conducted, adding 10 μL of CuCl_2_ to induce the fibril aggregation. Each experiment was composed of an assay and a blank sample, where the blank sample lacks Aβ_1-42_, to monitor the effect of the compounds on the fluorescence emission. The samples were left to incubate in a CERTOMAT WR water bath for 24 h at 37 °C with shaking. After 24 h, a Glycine-NaOH buffer (pH = 8.5) containing ThT were added to each sample in a microplate. The fluorescence signal was monitored at 446 nm (λ_exc_) and 486 nm (λ_em_) with a Varian Cary Eclipse fluorimeter. Amyloid β peptide (Aβ_1-42_) was purchased from GeneCust as a lyophilised powder and stored at −20 °C [12,13,14].

#### 3.3.4. Metal Complexation

##### Materials and Equipment

General analytical grade reagents were purchased from current suppliers and they were used without further purification. The aqueous iron(III) (0.0177 M), copper(II) (0.015 M) and zinc(II) (0.0156 M) stock solutions were prepared from 1000 ppm standards (Titrisol) and its metal content was evaluated by atomic absorption. The 0.1 M HCl solution used in calibration of the glass electrode was prepared from a Titrisol ampoule. The iron stock solution was prepared in an HCl excess, to prevent hydrolysis, and its exact concentration in HCl was accurately determined by the usual standard-addition method using 0.1 M HCl (Titrisol). The titrant used in pH-potentiometric and spectrophotometric titrations was prepared from carbonate free commercial concentrate (Titrisol, KOH 0.1 M ampoules). The KOH solution was standardized by titration with a solution of potassium hydrogen phthalate and was discarded whenever the percentage of carbonate, determined by Gran’s method [49] was greater than 0.5% of the total amount of base. The potentiometric studies were performed with an automated potentiometric apparatus containing a CrisonmicropH 2002 milivoltimeter, a Crison microBu 2031 burette and a Haake thermostatic bath (T = 25.0 ± 0.1 °C), controlled by PASAT program. The spectrophotometric titrations were done using a Perkin-Elmer Lambda 35 spectrophotometer.

##### Potentiometric and Spectrophotometric Studies

Titrations of the selected compound **1**, alone or in the presence of the metal ions, were accomplished in a 15% *w*/*w* DMSO/H2O medium, at *T* = 25.0 ± 0.1 °C and ionic strength (I) 0.1 M KCl, by using 0.1 M KOH as titrant. Both glass and Ag/AgCl reference electrodes were previously conditioned in different DMSO/H_2_O mixtures of increasing DMSO % composition and the response of the glass electrode was evaluated by strong acid–strong base (HCl/KOH) calibrations with the determination of the Nernst parameters by Gran’s method [49]. The measurements were performed in a final volume of 30.00 mL and the ligand concentrations (C_L_) were 6.7 × 10^−4^ M for the potentiometric studies and 4 × 10^−5^ M for the spectrophotometric titrations, under different C_M_/C_L_ (M = Fe, Cu, Zn) ratios: 0:1 (L), 1:1, 1:2 and 1:3. The spectrophotometric measurements were carried out in a 250–320 nm wavelength range at pH ca 2.5-8.0. Under the stated experimental conditions, the pKw value (13.6) was determined and subsequently used in the computations. The stepwise protonation constants of the ligands, *K*i = [H_i_L]/[H_i-1_L][H], and the overall metal–complex stability constants, β (M_m_H_h_L_1_) = [M_m_H_h_L_l_]/[M]^m^[H]^h^[L]^l^, were calculated by fitting the pH-potentiometric and spectrophotometric data with, respectively, Hyperquad 2008 [36] and Psequad [37] programs. The metal hydrolysis model was determined under the defined experimental conditions (I = 0.1 M KCl, 15% *w*/*w* DMSO/H_2_O, T = 25.0 ± 0.1 °C) and the following values of stability constants were included in the fitting of the experimental data towards the equilibrium models related to the Fe(III)/L, Cu(II)/L and Zn(II)/L systems: log β (FeH_-1_) = −1.79, log β (FeH_-2_) = −4.9, log β (FeH_-4_) = −20.0; log β (Cu_2_H_-2_) = −9.5; log β (ZnH_-1_) = −8.6, log β (ZnH_-3_) = −27.2, log β (Zn_2_H_-1_) = −8.6. The species distribution curves were obtained with the Hyss program [36].

#### 3.3.5. Cell Viability and Neuroprotection 

SH-SY5Y human neuroblastoma cells (ATCC-CRL-2266) were seeded in DMEM/F12 (Dulbecco’s Modified Eagle Medium/Ham’s F-12 Nutrient Mixture; Thermo Fisher Scientific) with 10% heat inactivated fetal bovine serum, 50 U/mL penicillin and 50 U/mL streptomycin under a humidified atmosphere with 95% relative humidity at 37 °C at a 0.16 × 106 cells/mL density. Stock solutions from tested compounds (1, 2, 4 and 5) were prepared in DMSO at a 25 mM concentration and stored at −20 °C until needed. In order to select the highest non-toxic concentration, a dose-response screening was performed, where SH-SY5Y cells were exposed to the compounds for 25 h. The selected concentrations for each compound were: 75 µM final concentration for 1 and 2; 10 µM final concentration for 4 and 20 µM final concentration for 5. The final concentration of DMSO did not exceed 0.05% (*v*/*v*) and no alterations on cells were observed. For the neuroprotection assays, the selected compounds were added to the cell media 1 hour prior to the incubation with Aβ_1-42_ or L-ascorbic acid/ferrous sulphate. Aβ_1-42_ or *L*-ascorbic acid/ferrous sulphate treatment was maintained for another 24 h. Aβ_1-42_ was prepared as 276.9 µM stock in sterile water and administrated in the medium at a 2.5 µM final concentration. Ferrous sulphate was freshly prepared as 0.36 M stock in water and added to the medium at 2.5 mM final concentration. L-ascorbic acid was freshly prepared as 80 mM stock in water and added to the medium at 5 mM final concentration. Aβ_1-42_ was purchased from Bachem (Torrance, CA, USA) and ferrous sulphate and L-ascorbic acid from Sigma Chemical Co (St. Louis, MO, USA).

Cell viability was measured by using the MTT (3-(4,5-dimethylthiazol-2-yl)-2,5-diphenyl tetrazolium bromide) reduction assay. This test is based on the presence of succinate dehydrogenase enzyme in viable cells, that allows them to metabolize MTT into a formazan. After treatment, the medium was removed, and cells were washed with 1x PBS one time to remove dead cells. 200 µL of MTT solution (0.5 mg/mL) was added to each well and incubated for 2 h protected from light. Formazan precipitates were then dissolved in DMSO and absorbance measured at 570 nm. The ability to reduce MTT was expressed as a percentage of untreated control cells.

##### Pharmacokinetics Properties

Pharmacokinetics properties were calculated using the software QikProp v.2.5 [46] for parameters clog P (lipophilic character), log BB (the capacity to cross the blood brain barrier), Caco2 permeability (the velocity of intestinal absorption), the activity in the CNS (central nervous system) and the verification of the Lipinski’s rule. To calculate these parameters, the optimized structures of the compounds obtained in Maestro [50] were submitted to the software QikProp v.2.5. Drug-likeness predictions were made with the SwissADME web service (http://www.swissadme.ch) [51].

### 3.4. Molecular Docking

Compounds **5**, with standard values of bond lengths and valence angles, were built within Maestro software package [50] as protonated skeleton, and passed to Open Babel [52] for a 10,000 steps of Steepest Descent minimization using the Universal Force Field. Chain A of eeAChE (PDB 1EVE) enzyme structure was passed to the Protein Preparation Wizard interface of Maestro, where water molecules were removed, and hydrogen atoms added, optimizing their position, and determining the protonation states of residues according to PROPKA prediction at pH 7.0. AMBER UNITED force field electrostatic charges [53] were loaded on the protein structure, while the molcharge complement of QUACPAC [54] was used in order to achieve Marsili-Gasteiger charges for the inhibitors. Affinity maps were calculated firstly on a 0.500 Å spaced 100 × 100 × 100 Å^3^ cubic box in the blind test dockings, and afterwards on 0.375 Å spaced 85 × 85 × 85 Å^3^ cubic box, centered on thedonepezil crystallized structures, and the accessibility of the binding site was exploited throughout 1000 runs of Lamarckian Genetic Algorithm (LGA) implemented in AUTODOCK 4.2.6 [55] using the GPU-OpenCL algorithm version [56]. Explicit water contribution was taken into account according to the hydration force field of AUTODOCK [57], and the population size and the number of energy evaluations figures were set to 300 and 10000000, respectively. Among all the plausible binding poses, the most similar pose using donepezil as query was selected by means of the shape matching algorithm ROCS [58].

## 4. Conclusions

Alzheimer’s disease is generally considered a multifactorial pathology. For this reason, a good amount of recent research effort has focused on the development of multi-target drugs. The main goal of this work was to design and prepare Tenuazonic acid (TA) derivatives (**1**–**3**) starting from the structure of the natural scaffold. Though the results obtained for AChE inhibition for compounds **1**–**3** are in the micromolar range and relatively similar to TA, these compounds present improved inhibition of Aβ aggregation, metal chelation capacity, potential oral absorbance and are non-cytotoxic towards SH-SY5Y human neuroblastoma cell line which makes them good building blocks for the development of future anti-neurodegenerative drugs. In fact, the selected compound **1** shows a good chelating ability towards copper, with desired selectivity as compared to zinc, and unsurprisingly but not uninterestingly, it is also a strong iron chelator, confirming to be a promising scaffold for the development of new multi-target molecules. Moreover, two hybrids with a donepezil-mimetic moiety (**4** and **5**) were also synthesized and showed significantly higher inhibition of AChE (IC_50_ 16–24 μM) and excellent inhibition of Aβ aggregation (72.0–78.4% at 40 μM), besides keeping the potential oral bioavailabilty. Molecular docking of compound **5** also showed how its length is comparable to that of Donepezil and highlighted the positive interactions that occur between the compound and the enzyme. Moreover, although there is no direct correlation between AChE inhibition and cell protection from Aβ toxicity, compound **5** seems to prevent Aβ aggregation itself. The fact that all the tested compounds are not *per se* cytotoxic on the human neuroblastoma cell line of choice is also a positive result that will contribute to further develop this class of potential therapeutic agents. 

All tested compounds, while not showing activity on all desired targets, still show activity on at least two of them, demonstrating they are all promising hit compounds for further drug design and development. Therefore, the newly developed compounds can be considered as an interesting starting point for the development of future multi-functional drugs potentially useful for the treatment of AD. 

## Figures and Tables

**Table 1:** Results from AChE inhibition, radical scavenging and inhibition of Aβ aggregation assays.

Compound	eeAChEi ^a^ IC_50_ (μM)	Antioxidant Activity EC_50_ (µmol/µmol of DPPH ± SEM) ^b^	Inhib. Aβ Aggreg.(%) ^c,d^	
			Self-Induced	Cu^2+^-Induced
**1**	42 ± 3	10.8 ± 0.2	32.9	36.6
**2**	57 ± 4	6.3 ± 0.1	12.4	28.2
**3**	44 ± 4	>30	57.4	63.8
**4**	16 ± 2	>30	72.0	-
**5**	24 ± 3	>30	78.4	-
**Tenuazonic acid**	65 ± 4 8.13 ± 0.08 [19]	- 2.6 ± 0.2 [19]	25.2 50 ± 8 ^e^ [19]	-
**Donepezil**	0.026 [13]	>30	-	-
**Tacrine**	0.11 ± 0.01	-	31.2	-

^a^ The values are the mean of three independent experiments ± SEM (standard error of the mean); AChE inhibition from electric eel; ^b^ EC_50_ values for the DPPH assay (Mean ± SEM of 3 independent experiments); ^c^ Inhibition of self-mediated Aβ_1-42_ aggregation (% at 40 μM) with or without copper (40 µM). The thioflavin-T fluorescence method was used and the measurements were carried out in the presence of an inhibitor (40 µM); ^d^ The values are the means of three independent measurements in duplicate (SEM < 10%); ^e^ *C*_inhibitor_ = 100 µM [19].

**Table 2:** Stepwise protonation constant of compound **1** and global formation constants ^a^ of its Fe(III), Cu(II) and Zn(II) complexes (*T* = 25.0 ± 0.1 °C, *I* = 0.1 M KCl, 15% *w*/*w* DMSO/water) and pM ^b^ values.

Compound	M_m_H_h_L_l_ (m,h,l)	log *K*	log β(Fe_m_H_h_L_l_)	log β(Cu_m_H_h_L_l_)	log β(Zn_m_H_h_L_l_)
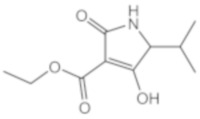 1	(011)	2.334(5) ^c^			
		2.85(4) ^d^			
	(101)		9.46(7) ^d^	4.08(8) ^d^	3.16(8) ^c^
	(1-21)		-	−4.15(7) ^d^	−11.75(5) ^c^
	(102)		14.30(7) ^d^	-	-
	(1-12)		-	6.56(6) ^d^	-
	(1-22)		5.83(8) ^d^	-	-
	(103)		19.4(1) ^d^	-	-
	**pM**		**16.6**	**11.6**	**6.0**

^a^ β_MmHhL1_ = [M_m_H_h_L_l_]/[M]^m^[H]^h^[L]^l^; ^b^ pM = −log[M] at pH 7.4 (C_L_/C_M_ = 10, C_M_ = 10^−6^ M) [38]; ^c^ potentiometric values; ^d^ spectrophotometric values.

**Table 3:** Pharmacokinetic properties of the compounds as predicted in silico by software QikProp v.2.5 [46].

Comp.	MW	clog *P*	log BB	Caco2 Permeability (nm/s)	CNS	Violations of Lipinski’s Rule of 5
**1**	213.233	1.662	−0.979	417	–	0
**2**	261.277	2.614	−0.931	525	–	0
**3**	185.179	0.862	−0.938	335	–	0
**4**	390.527	2.623	−0.128	19	+	0
**5**	342.483	1.327	0.070	19	+	0
